# Cyanotoxins and Food Contamination in Developing Countries: Review of Their Types, Toxicity, Analysis, Occurrence and Mitigation Strategies

**DOI:** 10.3390/toxins13110786

**Published:** 2021-11-06

**Authors:** Mohamed F. Abdallah, Wannes H. R. Van Hassel, Mirjana Andjelkovic, Annick Wilmotte, Andreja Rajkovic

**Affiliations:** 1 Department of Food Technology, Safety and Health, Faculty of Bioscience Engineering, Ghent University, 9000 Ghent, Belgium; andreja.rajkovic@ugent.be; 2 Sciensano, Chemical and Physical Health Risks, Organic Contaminants and Additives, Leuvensesteenweg 17, 3080 Tervuren, Belgium; wannes.vanhassel@sciensano.be; 3 Sciensano Research Institute, Chemical and Physical Health Risks, Risk and Health Impact Assessment, Ju-liette Wytsmanstreet 14, 1050 Brussels, Belgium; mirjana.andjelkovic@sciensano.be; 4 BCCM/ULC Cyanobacteria Collection, InBios-Centre for Protein Engineering, Université de Liège, 4000 Liège, Belgium; awilmotte@uliege.be

**Keywords:** cyanotoxins, microcystins, nodularins, cylindrospermopsin, food safety, developing countries, seafood, Africa, Asia, Latin America

## Abstract

Cyanotoxins have gained global public interest due to their potential to bioaccumulate in food, which threatens human health. Bloom formation is usually enhanced under Mediterranean, subtropical and tropical climates which are the dominant climate types in developing countries. In this context, we present an up-to-date overview of cyanotoxins (types, toxic effects, analysis, occurrence, and mitigation) with a special focus on their contamination in (sea)food from all the developing countries in Africa, Asia, and Latin America as this has received less attention. A total of 65 publications have been found (from 2000 until October 2021) reporting the contamination by one or more cyanotoxins in seafood and edible plants (five papers). Only Brazil and China conducted more research on cyanotoxin contamination in food in comparison to other countries. The majority of research focused on the detection of microcystins using different analytical methods. The detected levels mostly surpassed the provisional tolerable daily intake limit set by the World Health Organization, indicating a real risk to the exposed population. Assessment of cyanotoxin contamination in foods from developing countries still requires further investigations by conducting more survey studies, especially the simultaneous detection of multiple categories of cyanotoxins in food.

## 1. Introduction

Cyanotoxins are secondary metabolites produced by certain toxic species of cyanobacteria, a morphologically diverse group of Gram-negative photosynthetic bacteria, which are also known as blue-green algae [1,2,3]. These cyanotoxin-producing bacteria negatively affect ecosystems as they colonize a wide range of niches in aquatic and terrestrial environments [4]. Furthermore, human exposure to toxic cyanobacteria and/or their toxins results in a series of mild to life-threatening health implications [5,6,7]. The problem of cyanotoxin production, due to the formation of toxic algal blooms (cyano-blooms) or scums or mats, and the subsequent risks for human and animal health are considered a subject of concern all over the world, especially when we consider the massively increasing anthropogenic activities and global warming issues [8,9,10,11,12]. This is due to a progressive eutrophication which induces an excessive growth of algae and enhances the formation of cyanotoxins [13]. In addition, global warming is anticipated to raise the frequency of blooms and their duration. Therefore, toxic cyanobacterial blooms can be recognized as a global emerging environmental threat. The relationships between the environmental factors (such as nutrient concentration, sunlight intensity, water temperature, water turbidity, pH, salinity, carbon availability, and water stratification and flow) and the proportion of the cyanotoxin-producing bacteria in relation to other non-toxigenic cyanobacteria as well as the amount of free and/or cell-bound toxins are yet to be fully elucidated, which represents a knowledge gap [13,14,15].

Human exposure to cyanotoxins is likely to occur through direct consumption of toxin-contaminated drinking water or seafood such as fish, mussels, and crustaceans that ingested toxigenic cyanobacteria and/or their toxins i.e., there is a potential transfer through the food web [5,16,17,18,19,20]. Uptake of cyanotoxins by agricultural plants, including the edible ones, following the irrigation with toxin-contaminated water has been also documented as a potential source of human exposure [16,21,22,23,24,25,26]. Codd et al. studied the uptake and accumulation of three different hepatoxic cyanotoxins in lettuce (*Lactuca sativa*) during their growth stage, while Crush et al. studied the same phenomena in other edible plants such as ryegrass (*Loliumperenne*), clover (*Trifoliumrepens*) and rape (*Brassica napa*) in addition to lettuce and exposed them to 10 different hepatic cyanobacterial toxins [27,28,29]. Rice (*Oryza sativa*) was shown to accumulate cyanotoxins in different parts, including grains. However, the available data revealed that the studied cyanotoxins were detected in very low levels in the grains which may not be of concern to humans [30]. Although oral exposure is the main route, dermal exposure may occur, especially in recreational sport and other professional water activities (i.e., fishing), causing dermatitis and mild to severe allergic reactions or maybe a systemic toxic effect. In 2007, a young man was immersed by accident in an intense bloom in Salto Grande Dam, Argentina. A few hours later, general non-specific symptoms (nausea, abdominal pain, fever) were observed while other more serious symptoms (dyspnea, respiratory distress) appeared a few days later [31]. In 2015, there was a reported case of a 20-month-old child suffering from general gastroenteritis manifested by diarrhea, vomiting, fatigue, and jaundice. The history of the case indicated recreational activities (Carrasco and Malvín beaches, Uruguay) with her family a few hours before the appearance of the symptoms. A liver function test indicated acute liver failure and the child was subjected to liver transplant surgery and the chemical analysis of a sample from her liver showed the exposure to hepatoxic cyanotoxins [32].

Exposure via inhalation of dusts containing terrestrial toxic cyanobacteria is unlikely to occur, but cannot be excluded or ignored [4,33]. Other accidental exposure may also happen by other routes. In 1996, in Brazil, 52 persons died out of 130 persons subjected to renal hemodialysis treatment after accidental water contamination with two groups of hepatotoxic cyanotoxins [34,35,36]. Besides the exposure route, other parameters such as dose, type of cyanotoxins, co-exposure to multiple toxins/chemicals and the health status of the exposed organism are crucial for the toxic outcome(s) [2,4].

The current review provides an up-to-date overview on different research aspects of cyanotoxins covering their types, toxic effects, analytical techniques for detection, and occurrence in (sea)food. The main core of the current work is focusing on cyanotoxin contamination in (sea)food from all the developing countries in Africa, Asia, and Latin America as it has received less attention. The last part of the review highlights different mitigation strategies and the current knowledge gap that can be considered as future perspectives.

## 2. Common Cyanotoxins: Classification, Accumulation, and Toxicity

Cyanotoxins are quite diverse in their physico-chemical properties and toxicity. There are several approaches/ways for classification or categorization of cyanotoxins depending on their chemical structures (cyclic peptides, alkaloids, and lipopolysaccharides) or their producing bacterial species (*Dolichospermum*, *Aphanizomenon*, *Cylindrospermopsis*, *Lyngbya*, *Microcystis, Nostoc*, *Oscillatoria* etc.) [35,37]. Another way for grouping cyanotoxins is based on their toxicity mechanism and the primary target organs affected in the body. Cyanotoxins are classified into hepatotoxins (e.g., microcystins, cylindrospermopsin, and nodularins), neurotoxins (e.g., anatoxin-a, anatoxin-a(s), saxitoxins), and dermatotoxins or irritant toxins (e.g., lipopolysaccharides, lyngbyatoxin, aplysiatoxins) [4,38,39]. In general, concentrations of cell-bound (i.e., intra-cellular) toxins are several orders of magnitude higher than for the cell-free (i.e., extra-cellular) toxins as fresh or brackish water dilute the produced toxins. The biological importance of cyanotoxins produced in cyanobacteria still is not completely unraveled. They could be produced as a biological weapon against zooplankton and protozoa so that the grazing rate is reduced [1,15,40,41]. In the following section, a brief description is given for the common cyanotoxins according to their chemical structure with some known and updated facts concerning their producing bacteria, occurrence, toxicities and accumulation in food.

### 2.1. Toxic Cyclic Peptides

#### 2.1.1. Microcystins

**Figure 1:**
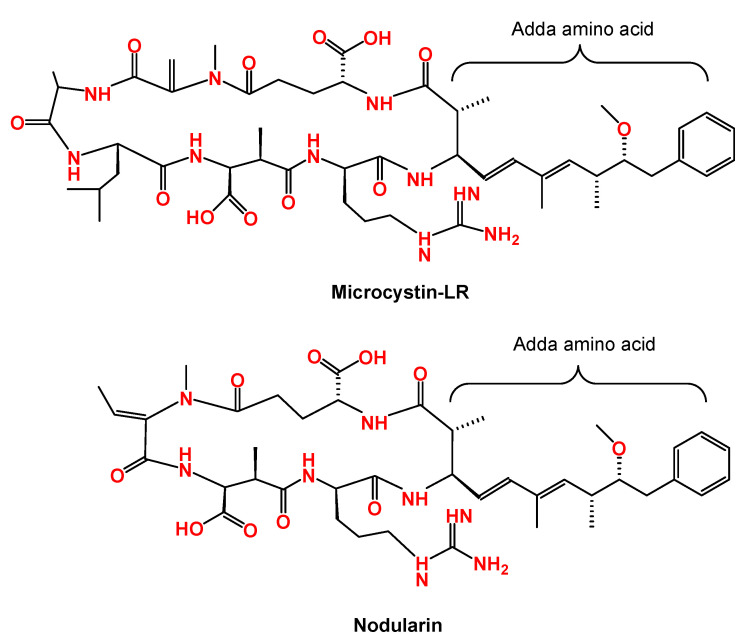
Chemical structure of microcystin-LR and nodularin.

Microcystins (MCs) are the most investigated group of the cyanobacterial toxins due to their widespread occurrence in freshwater lakes, ponds, rivers, estuaries, and coastal waters all over the world [14,42]. MCs are produced by various species of some cyanobacteria genera (*Microcystis*, *Aphanizomenon*, *Dolichospermum* (ex *Anabaena*), *Nostoc*, *Limnothrix*, *Phormidium*, *Oscillatoria*, and *Planktothrix*) [1,4,5,43]. The first isolation of MCs was from the cyanobacterium *Microcystis aeruginosa* and the full structural identification of the first MCs congeners was obtained in 1984 [3,19,44]. Later, other species were reported to produce MCs, such as *Planktothrix* and *Dolichospermum* species, and until now there are more than 30 species that have the capability of producing MCs [45]. Optimal environmental conditions (such as water stability, light intensity, CO_2_ availability, temperature, and pH) for bloom formation and/or production of MCs are different among the cyanobacterial species [14]. MCs are non-ribosomal processed cyclic heptapeptides and distinguished by the presence of the amino acid Adda ((2S,3S,8S,9S)-3-amino-9-methoxy-2,6,8-trimethyl-10-phenyl-4,6-decadienoic acid) in their structure [5,45]. As a result of multiple combinations of different amino acids with the presence of various changes in other functional groups, more than 279 variants (or congeners) have been described so far [44]. The molecular weights of these variants range between 881 and 1360 Da [44]. Among these, the most common three congeners are originating from the presence of three amino acids, leucine (L), arginine (R) or tyrosine (Y) in positions two and four of the cyclic chain to form MC-LR, MC-RR and MC-YR [38,42,46]. The most studied structural variant which is also considered the most potent on the basis of its acute toxicity is MC-LR (Figure 1). This toxin possess leucine (L) at position two and arginine (R) at position four [3].

**Figure 2:**
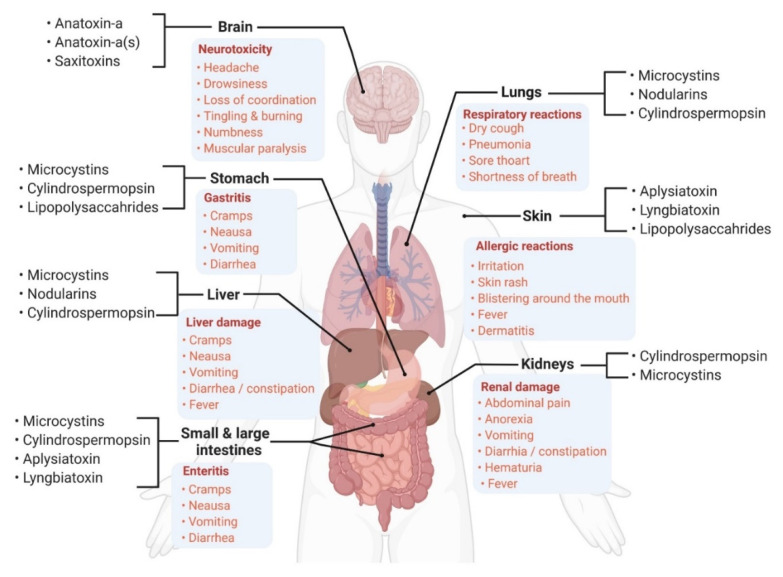
Possible toxic effects of cyanotoxins and clinical symptoms in humans.

The mechanism of toxicity is based on the inhibition of protein phosphatase types 1 and 2A, which leads to hyperphosphorylation of cellular proteins in the liver causing hepatoxicity, liver damage, massive hemorrhage and death [43,46]. However, other organs such as the gastrointestinal tract, kidney, lung, and heart are also considered as target organs for MCs toxicity (Figure 2) [43,45,47,48]. Acute cases of MC-LR poisoning have caused several animal and humans deaths in several countries, mainly through drinking of contaminated raw water [34,35,36,42,49].

MCs mainly accumulate in the liver of the exposed fish and other aquatic organisms [18,19,50], which does not represent a real concern for humans since these parts are usually non-edible [16]. MCs can also accumulate in other organs [6], and they can even be transferred into the offspring in some invertebrates [51]. Indeed, MCs accumulation in fish muscles and in other invertebrates can lead to human intoxication [17,52,53,54,55]. Such accumulation happens in case of high level of exposure when the pre-systematic hepatic elimination is overwhelmed and cannot tolerate more MCs. No clear correlation could be found between the detected concentrations in the liver or viscera and muscles. The failure to find a clear correlation is related to the high variability of the results in different species of fish and other factors related to feeding strategies. Until now, it is unclear whether MCs can be transferred to milk since the available data rely only on a very limited number of animals from only two studies available in the literature [27]. In its external scientific report, the European Food Safety Authority (EFSA) suggested that more precise analytical methods should be used/developed which take into account the bound-MCs, and not only the free ones. There is an ongoing debate, based on contrasting results, on the effect of cooking on MCs concentrations, as these compound are very stable and non-volatile [16].

For plants, irrigation with MCs-contaminated water causes growth inhibition in numerous plant species with a possibility of translocation and accumulation in their edible parts [26,29,38,56,57,58]. For instance, tomato (*Solanum lycopersicum*) and peppers (*Capsicum annuum*) bioaccumulate MC-RR in fruit and seed parts when they are irrigated with contaminated water from Lake Amatitlán, Guatemala [24]. A notable conclusion from these studies is that the bioaccumulation process occurs in a concentration-dependent manner [22,25]. Recent meta-analysis has shown that the bioconcentration of MCs in leafy vegetables is nearly three times higher than in other plants [26]. Furthermore, MC-LR elimination in lettuce may take up to 29–37 days as shown in a depuration kinetics study [58]. MC-LR has been classified as a possible hepatocarcinogenic agent (group 2B) by the International Agency for Research on Cancer (IARC) [59]. A correlation has been shown between the incidence of cyanobacterial blooms and gastrointestinal and prostate cancers in some studies from China. However, further investigations with a consideration of other confounding factors are required to confirm such a link [60,61,62,63].

#### 2.1.2. Nodularins

Nodularins (NODs) are another group of cyclic pentapeptides of cyanotoxins that cause hepatotoxic effects through inhibiting protein phosphatase 1 and 2A [14,64,65]. In fact, NODs (molecular weight of 825 Da) is a truncated MCs, based on the sequence of the gene cluster responsible for its synthesis. Currently, there are 10 variants of the NODs family based on a unique part of their structures, which is the amino acid Adda, (2S,3S,8S,9S)-3-amino-9-methoxy-2,6,8-trimethyl-10-phenyldeca-4,6-dienoic acid (Figure 1) [2,3,65]. Since NODs and MCs share similar characters such as polarity and chemical structure, they are often included in one analytical detection method [1,2]. Unlike MCs, the NODs family is mostly found in cyanobacterial blooms from brackish waters. NODs are usually produced by the cyanobacterium *Nodularia spumigena* and the benthic species *Nodularia sphaerocarpa* [1,7,64]. Higher temperature and phosphorus concentrations stimulate more production of intracellular NODs [35]. In the last three decades, the toxin-producing bacteria and NODs (especially Nodularin-R, the most common variant) have been detected in different parts of the world such as Australia, the Baltic Sea, North Europe, and the USA. There is no clear evidence of the bioaccumulation of NODs in crops even under lab scale condition. A very few reports documented the occurrence of NODs in the developing countries [66], and there is no published case of human poisoning due to NODs exposure. Animal deaths from the developing countries are described in the literature [67]. As there is not enough toxicological data for NODs, no regulations have been established in any seafood [65]. NODs are classified as non-carcinogenic substances to human (group 3) by IARC [59].

### 2.2. Toxic Alkaloids

#### 2.2.1. Anatoxin-a and Anatoxin-a(s)

The most prominent member of the cyanobacterial toxic alkaloids is anatoxin-a (ANTX) which is mainly produced by *Dolichospermum* sp. (*a.o. D. planctonica*, *D. flos-aquae*, *D. spiroides*, *D. circinalis*), *Oscillatoria* sp., *Cylindrospermum* sp., and *Aphanizomenon* sp. [1,10]. The toxin is a secondary bicyclic amine alkaloid (molecular weight of 165 Da) and has been detected in environmental samples from several parts in the world including the developing countries [39,68,69]. ANTX was the first cyanotoxin described with neurotoxic effects in experimental and wild animals [19]. There is one (regularly) reported variant of ANTX called homoanatoxin-a, which is a phosphate ester with a cyclic N-hydroxyguanidine structure and has a molecular weight of 179 Da. ANTX binds irreversibly to the nicotinic acetylcholine receptors, and symptoms of toxicity are manifested by muscle fasciculations, abdominal breathing, cyanosis, convulsions, and death due to paralysis of the respiratory muscles and asphyxia [10]. Other possible toxic effects and symptoms of ANTX toxicity in humans are summarized in Figure 2. There are few recorded reports on ANTX epidemics in animals from developing countries. In Kenya, mass mortalities in Lesser Flamingos happened due to the exposure to ANTX with two other variants of MCs (MC-LR, MC-RR) [68]. The maximum detected concentrations of ANTX in the liver samples of dead birds were six times higher than concentrations of MCs (up to 0.93 µg MC-LR eq/g fresh weight).

**Figure 3:**
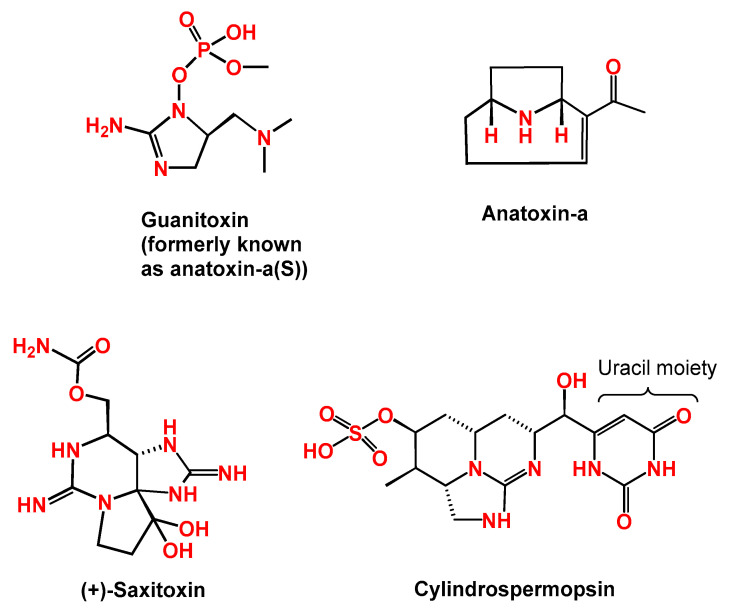
Chemical structure of guanitoxin, anatoxin-a, saxitoxins and cylindrospermopsin.

Anatoxin-a(s) (ANTXs) is another neurotoxic alkaloid with molecular weight of 252 Da. ANTXs is a N-hydroxy guanidine methylphosphate ester causing irreversible inhibition of acetylcholinesterase similar to organophosphate compounds such as paraoxon [10,39]. ANTXs was tested in male mice after intraperitoneal injection, which led to excessive salivation, dyspnea, cyanosis, and seizures before death due to respiratory arrest [70]. It was estimated that the neurotoxicity of ANTXs is ten-folds higher toxicity than ANTX. Recently, researchers have proposed renaming the toxin to guanitoxin since it has unrelated chemical structure, mechanism of action, and biosynthesis (Figure 3) [71].

#### 2.2.2. Cylindrospermopsin

Another member of the alkaloid family is cylindrospermopsin (CYN), a tricyclic guanidine moiety combined with hydroxymethyl uracil (molecular weight of 415 Da) (Figure 3) [72]. The toxin has two congeners called: 7-epi-CYN and 7- deoxy-CYN. Various species of cyanobacteria are able to produce CYN such as *Cylindrospermopsis*, *Aphanizomenon*, *Dolichospermum*, *Lyngbya*, *Rhadiopsis, Planktothrix*, and *Umezakia* [73,74,75]. The first report on the presence of CYN-producing cyanobacterium, *Cylindrospermopsis raciborskii*, was more than 120 years ago in Indonesia. After that, researchers have documented its occurrence in Brazil, Egypt, India, Mexico, Senegal, and Thailand. However, the toxin itself was chemically defined from *C. raciborskii* in 1992 [74,76,77,78,79,80]. Since then, this bacterial species, which is still a major producer of the toxin, has been reported in both brackish and fresh water from tropical and subtropical regions [73,74,79]. Bioaccumulation of CYN in various aquatic animals and plants from developing countries has been reported [19,79,81,82,83]. Recently, CYN translocation from spinach roots to leaves has been observed under controlled experimental conditions for 21 days in a CYN-doped nutrient medium [56]. The study conducted by Corderio-Araújo et al. measured the bioaccumulation and depuration of CYN in lettuce. Interestingly, both bioaccumulation and depuration decreased with increasing the exposure concentration [84].

Unlike MCs, CYN toxin is mainly present as an extra-cellular or free toxin (up to 90% of total CYN) rather than as an intra-cellular metabolite. The toxin is highly soluble in water, very stable and persistent in aquatic environments, and resistant to some standard water treatment processes [85,86]. CYN exerts multiple toxicities which may include cytotoxicity, hepatotoxicity, genotoxicity, neurotoxicity, and immunotoxicity [7,75,87]. Possible toxic effects and clinical symptoms resulting from CYN exposure are depicted in Figure 2. The mechanism of its toxicity is mainly mediated through an irreversible inhibition of glutathione, protein synthesis, and cytochrome P450 leading to apoptosis [27,82,88]. As the information on CYN carcinogenicity is not enough, the toxin is classified as a non-carcinogenic agent by IARC [59].

#### 2.2.3. Saxitoxins (STXs)

Saxitoxins (STXs), a family of more than 57 congeners or analogs, are another class of natural alkaloids that pose potent rapid neurotoxic activities and maybe death within few hours [10,89]. Blocking the voltage-gated sodium channels of the neurons which leads to muscular paralysis is the main toxic mechanism [90,91]. STXs are synthesized by some cyanobacteria of the genera *Dolichospermum*, *Aphanizomenon*, *Cylindrospermopsis*, *Lyngbya*, *Planktothrix*, *Raphidiopsis*, *Fischerella*, *Geitlerinema* and *Scytonema* [39]. However, they are produced mainly by marine microalgae, as well as a few calcareous red macroalgae [90,92]. STXs are collectively known as paralytic shellfish toxins (PST) or Poisons [91]. Until now, no human deaths or poisoning due to the presence of cyanobacterial STXs in freshwater environments have been reported. However, a worldwide distribution of PST is documented based on the consumption of bivalves and other shellfish contaminated with STXs from other sources [19,93]. Possible symptoms of STXs-related neurotoxicity, in the case of exposure, may include headache, mouth tingling, numbness of extremities, difficulty in breathing, and muscular paralysis (Figure 2). Experimental work and survey studies have been carried out to investigate the bioaccumulation of STXs in freshwater Nile tilapia (*Oreochromis niloticus*) [93] and other marine organisms [81,94,95]. The results revealed the ability of STXs to accumulate, during toxic cyanobacterial blooms, inside the muscles or edible parts which indicates possible human exposure to STXs via ingestion of contaminated seafood.

### 2.3. Lipopolysaccharides

Lipopolysaccharides (LPS) are known to form the outer layer of all cyanobacterial wall, as they belong to Gram-negative prokaryotes [7,96]. LPS are quite diverse in their structure among cyanobacterial strains due to phylogenetic differences. A unique feature of cyanobacterial LPS is the lack of heptose and 3-deoxy-d-manno-octulosonic acid, which is present mainly in other Gram-negative LPS. Production of LPS is reported in many cyanobacterial species such as *Anacystis nidulans*, *Microcystis aeruginosa*, *Dolichospermum* sp., *Spirulina platensis* and *Oscillatoria* sp. [13,97]. Human contact with LPS, which often happens during recreational activities, leads to some inflammatory reaction of the skin and probably some gastrointestinal discomfort due to gastro-enteritis (Figure 2) [19,98]. Therefore, they are also called endotoxins or irritant toxins. Until now, the mechanism of LPS toxicity has not been well studied and their toxicity is hypothesized to be linked with some host-mediated factors. In addition to PLS, there are other natural toxins that cause similar signs in humans such as lyngbyatoxin and aplysiatoxins, which are also thought to be tumor-promoting molecules [3,37].

## 3. Analysis of Cyanotoxins in Food

Past and current techniques for (semi)quantitative analysis of cyanotoxins are based on two approaches. Chemical analytical methods which include capillary electrophoresis (CE) and liquid chromatography (LC) coupled with ultraviolet detector (UV) or photodiode array detector (PDA) or fluorescence detectors (FLD) or tandem mass spectrometry (MS/MS) [99,100,101] while the biological assays implies mouse bioassays, enzyme-linked immunosorbent assay (ELISA), protein phosphatase inhibition assays (PPIA) [42,65,102]. A general comparison between these techniques is outlined in Table 1. Most of these techniques have already been used to detect cyanotoxins in food matrices from the developing countries (shown in the following sections). LC-MS/MS has become the most popular technique as the developed methods are reliable, selective, and sensitive, rendering them more suitable for large-scale studies. Although validated analytical methods for the quantitative detection of cyanotoxins in different food matrices exist, many published papers focused on MCs [103,104,105,106,107]. Few studies considered the simultaneous detection of few MCs variants with other cyanotoxins such as: MCs and NODs in fish and algal supplement [99,108,109]; MCs and CYN in lettuce [110] and mussels [111]; and MCs, NODs, ANTX, CYN, and SXTs in fish tissue [112]. Like other natural toxins such as mycotoxins, some cyanotoxins as MCs can be found either in a free or bound form. Bound MCs are usually formed as a result of detoxification inside animal bodies and plants after a conjugation process with several conjugates (glutathione, cysteine, glycine, cysteine), resulting in what can be called modified cyanotoxins. This modification in the chemical structure allows them to escape normal routine analysis [113]. However, the bound compounds contribute to the overall toxicity and should be considered in the analysis and risk assessment.

Indeed, advanced analytical techniques can be optimized to detect a wide array of parent variants and their modified forms in several food matrices. Furthermore, such techniques can be used to develop reliable, simple, sensitive and cost-effective analytical approaches to cover multiple categories of cyanotoxins. Recently, high-resolution (UP)LC-MS methods have been developed for the detection of multiple classes of cyanotoxins in freshwater [100,114,115] and some algal dietary supplements [116]. These methods can be also used for a retrospective analysis of potential unknown cyanotoxins for future research [117]. On the other hand, developing portable devices for real-time on-site detection of MC-LR in water samples has been achieved using novel techniques such as fiber optical chemiluminescent biosensors [118]. These novel approaches offer a fast, easy, simple, and economic detection procedure of a single compound at low concentrations. The reported studies and developments of electrochemical affinity biosensors for cyanotoxins have been critically reviewed in the literature [119].

## 4. Occurrence of Cyanotoxins in Food from Developing Countries

**Figure 4:**
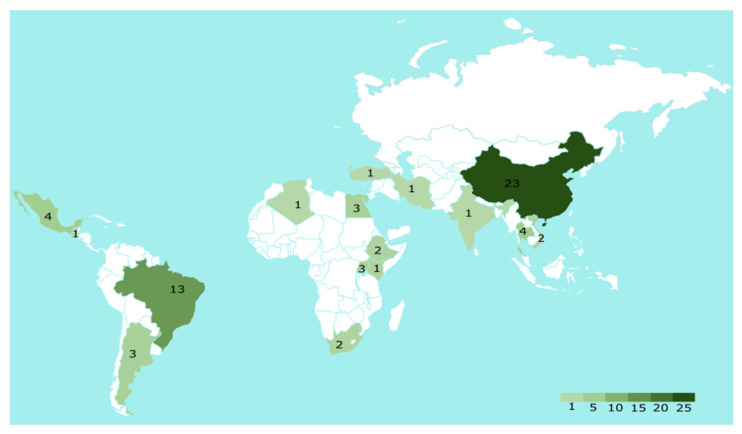
Geographical heatmap showing the conducted research (from 2000 until October 2021) on cyanotoxins in food (seafood and edible plants) from the developing countries in Africa, Asia, and Latin America. Countries with white color are lacking data. Developing countries are defined according to their Gross National Income (GNI) per capita per year, as calculated by the World Bank Atlas method, 31 October 2020.

An overview of the research conducted during the period between 2000 and October 2021 on cyanotoxins in (sea)food samples from developing countries is depicted in Figure 4. Data were obtained from indexed databases (e.g., Web of Science, ScienceDirect, Google Scholar), as well as from non-indexed bases (e.g., non-indexed articles). A total of 65 publications have been found reporting the contamination by one or more cyanotoxins in different food (60 papers on seafood and five papers on edible plants). The identified publications in Africa, Latin America, and Asia account for 18.5%, 32.3%, and 49.2% of the total number, respectively. From these papers, a total of 11 papers (including four papers on edible plants) were excluded as they either reported experimental work or did not focus on the natural occurrence of cyanotoxins. The rest of the identified papers (54 papers including one paper on edible pant) were considered for a detailed discussion in the following sections.

In general, only China and Brazil have conducted more research on cyanotoxin contamination in food in comparison with other developing countries from Africa, Asia, and Latin America. However, most of these investigations focused on MCs. Apart from these two countries, few countries have limited data on cyanotoxins. Furthermore, most of the developing countries in the three continents lacks data on the occurrence of cyanotoxins, especially in Africa. This represents a great challenge to enhance the food safety in these parts of the world. The supplementary data (S1, S2, and S3) contain an overview of all the research conducted within the same period (from 2000 until October 2021) on cyanotoxin occurrence in different water sources and/or (sea)food from the developing countries from each continent per year.

### 4.1. Occurrence of Cyanotoxins in Seafood from the Developing Countries of Africa

In general, most of the research conducted on cyanotoxins in Africa has focused on MCs, while other toxins were not under the spotlight. For cyanotoxins in seafood, all the identified survey studies were mainly targeting total MCs or MC-LR. A total of 12 publications have been found in the literature representing 18.5% of all the papers which have discussed the natural occurrence of cyanotoxins in food matrices (seafood only). One paper was excluded as it analyzed cyanotoxins in the liver only, which is a non-edible part for humans [120]. The collected data are mainly from a few African countries in the north (Algeria, Egypt), in the east (Ethiopia, Kenya, Uganda), and in the south (South Africa). The rest of the African countries lack occurrence data on cyanobacteria and their toxins in food. More data are available on the occurrence of MCs and other cyanotoxins from the analysis of blooms and scums present in lakes, reservoirs, rivers, and dams in a larger number of African countries (Figure S1, Table S1). So far, there are no available data on cyanotoxin contamination in bivalves or other crustacean edible aquatic organisms.

Nile tilapia was the most researched species in Africa (Table 2). One study analyzed MCs in *O. niloticus* from fish ponds with blooms located in the southern part of Egypt and found that MCs concentrations in muscles were several times above the equivalent of the provisional TDI, however, the number of samples was not mentioned [52]. The only study considering the bound or modified MCs was recently published, which indicated a very high level of exposure to humans, as the concentrations of bound MCs were thousands of times higher than those of free MCs [50]. The study also highlighted the high resistance of the investigated fish species, *O. niloticus*, as no mortalities were observed according to the authors. The same species was also surveyed from two different lakes in Uganda and high levels of MCs contamination were reported in the muscles in which MC-RR was more dominant followed by MC-LR and MC-YR [121]. However, another study surveyed the same species, in the same year (year of sampling) and sampling area, and found half of these levels for MC-RR [122]. The author also reported the same order of occurrence for MCs variants as the previous study.

Common carp (*Cyprinus carpio*) muscles can be a source for MCs exposure to Algerian consumers as the measured levels from a 1-year survey study were above the provisional TDI [123]. A drawback is that the study used phosphatase type 2A (PP2A) inhibition assay for the quantification of MCs and LC/MS-MS for the identification of MC variants only. Table sized fish of three species (Nile tilapia, common carp, African sharp tooth catfish) from Ethiopia were analyzed for MCs and CYN contamination. Although all the edible parts (i.e., muscles) were not contaminated, liver samples showed contamination with three MCs variants [124]. In a large-scale survey study, three tropical lakes (including Murchison Bay and Napoleon Gulf of Lake Victoria) and four smaller lakes (George, Mburo, Nkuruba, Saka) in Uganda showed that the analyzed samples from 19 different fish species were contaminated with MCs [53]. However, there was a significant variability due to differences in diet, species and seasons. The study concluded that consumption of some species of small size poses high risk, as they had high MCs concentration, while for others such as Nile perch (*Lates niloticus*) there was no risk [53]. Another study has also investigated the MCs contamination in small fish species, such as *Rastrineobola argentea,* which are known to be a popular food for local Kenyan consumers around Victoria Lake. Chemical analysis of MCs showed that MC-YR was present in 25% of the samples. This later study is the first in a semi-processed fish for human consumption which suggested that drying fish under sunlight might be a useful tool to reduce MCs content [125]. During a decomposing cyanobacterial bloom in the Loskop Dam area located in South Africa, 20 rednose labeo (*Labeo rosae*) and six Mozambique tilapia (*Oreochromis mossambicus*) samples were analyzed for MC-LR contamination and the results showed that the exposure levels to MC-LR toxin could be four times higher than the WHO guidelines [126].

The only study reported CYN was in collected commercial *O. niloticus* from three fishponds in southern Egypt in a range above the recommended guidelines by a factor of 1.3 to 14 times during summer and fall seasons due to the growth of bacterial bloom [79]. The authors estimated the risk based on a proposed TDI value of 0.03 μg/kg/day, however it is not an official guideline (proposed by Humpage and Falconer [127]). ANTX was detected in a cyanobacterial bloom from two freshwater lakes in Kenya almost 20 years ago [68,128]. However, there are no reports on its contamination in any seafood.

**Table 2:** Occurrence of cyanotoxins in seafood from the developing countries in Africa.

Country	Location/Year	Detected Toxins and Concentration Range (μg/kg, DW or WW)	Matrix	N	P	Method of Detection	References
Algeria	Lake Oubeir/2010–2011	MCs (329–680, DW)	*Cyprinus carpio* (muscles)	36	36	PP2A inhibition assay	[123]
Egypt	Sohag city/2000	MCs (45.7–102, WW)	*Oreochromis niloticus* (muscles)	NM	NM	ELISA	[52]
	Sohag city/2012–2013	CYN (19–280, WW)	*Oreochromis niloticus* (muscles)	99	63	ELISA	[79]
	Sohag city/2012–2013	Free MCs (0.02–0.38, WW); Bound MCs (15,000–19,000, WW)	*Oreochromis niloticus* (muscles)	198	NM	ELISA & Lemieux oxidation reactions and LC-PDA	[50]
Ethiopia	Lake Hora-Arsedi/2015	MCs <LOD	*Oreochromis niloticus*; *Tilapia zillii* (muscles)	8	Not detected	LC-HRMS	[129]
	Addis Ababa/2015–2016	MCs <LOD	*Oreochromis niloticus*; *Cyprinus Carpio*; *Clarias gariepinus* (muscles)	36	Not detected	LC-HRMS	[124]
Kenya	Nyanza Gulf, Lake Victoria/2011-2012	MC-YR (8–20, DW)	*Rastrineobola argentea* (whole small fish)	16	4	LC-MS/MS	[125]
South Africa	Mpumalanga province/2012	MC-LR (0.17–0.19, WW)	*Labeo rosae*; *Oreochromis mossambicus* (muscles)	26	21	ELISA	[126]
Uganda	Lake Mburo and Murchison Bay, Lake Victoria/2004–2005	MCs (5–121, WW)	*Oreochromis niloticus* (muscles)	72	51	LC-MS/MS	[122]
	Lakes (Victoria, Albert, Edward, George, Mburo, Nkuruba, Saka)./2007–2009	MCs (0.5–1917,WW)	19 species of fish (muscles)	399	NM	ELISA	[53]
	Lake Mburo & Murchison Bay, Lake Victoria/2004–2005	MCs (9.6–208.6, WW)	*Oreochromis niloticus* (muscles)	24	17	LC-MS/MS	[121]

DW, dry weight; WW, wet or fresh weight; N, number of samples; P, positive samples; LOD, limit of detection; NM, not mentioned; MCs, microcystins; MC-LR, microcystin-LR; MC-YR, microcystin-YR; CYN, cylindrospermopsin; PP2A, protein phosphatase type 2A inhibition assay; ELISA, enzyme-linked immunosorbent assay, LC-PDA, liquid chromatography with photodiode array detector; LC-MS/MS, liquid chromatography tandem mass spectrometry; LC-HRMS, liquid chromatography high-resolution mass spectrometry.

Biomarkers of exposure for 13 cyanotoxins were evaluated in Tanzanians living in the Ukerewe District in Mwanza. Multiple cyanotoxins (CYN, NODs, several congeners of MCs) were detected in their serum at variable concentrations [130]. More importantly, users of lake water were reported to suffer from some symptoms of acute illness such as sore throat and gastroenteritis, which were absent in pipe water users [131]. However, this does not exclude the need to implement an operational monitoring program for MCs without waiting for an outbreak or public illness to protect the public from potential exposure to cyanotoxins, especially MCs.

### 4.2. Occurrence of Cyanotoxins in Seafood from the Developing Countries in Asia

Our search in the literature, from different databases, has identified a total of 32 publications between the period of 2000 and October 2021, which counts for 49.2% of all the papers that investigated the contamination of cyanotoxins in (sea)food from the developing countries in Africa, Asia, and Latin America (Figure 4). Five papers were excluded as four papers either analyzed cyanotoxins in non-edible parts or did not mention the detected concentrations, while one paper discussed bioaccumulation of MCs congeners in a soil-plant system. All the remaining papers from Asia focused on different aquatic species which are consumed as seafood. Most of these papers (71.8%) were conducted in China and focused on MC-LR and/or -RR. Apart from that, only sporadic reports from Thailand, Vietnam, India, Iran, and Turkey are documented in the literature, which mainly studied the occurrence of MC-LR in seafood. Indeed, data on the occurrence of cyanotoxins from the analysis of environmental samples such as water or blooms and scums are more than those present on food (Figure S2, Table S2).

Although a considerable number of published papers are from China, in comparison to other Asian developing countries, most of these papers surveyed only two lakes (Taihu and Chaohu) (Table 3). Seasonal changes and distribution patterns in MCs (-LR, -YR and -RR) concentrations were studied in commonly consumed Chinese shrimps (*Palaemon modestus* and *Macrobrachium nipponensis*), silver carp (*Hypophthalmichthys molitrix*), bighead carp (*Aristichthys nobilis*), four freshwater bivalves, and mussels (*Corbicula fluminea*) collected from Lake Taihu and Lake Chaohu [51,132,133,134,135,136,137]. All these studies have reported considerable seasonal variations (from June to December of the studying years) and correlation with MCs with cyanobacterial bloom. In general, the detected levels of MCs, during cyanobacterial bloom periods, in muscles and other edible parts, in case of high consumptions, were above the permissible limit of MC-LR (Table 2). Furthermore, around 16%, 25%, 31% and 54% of the samples analyzed of silver carp muscles, bighead carp muscle, shrimp muscle, and mussels foot exceeded the provisional WHO tolerable daily intake level, respectively [51,133,134,135]. The authors also observed that sliver carp appeared to accumulate MCs in their muscles less than other animals [133], while bighead carp were strongly resistant to MCs [135].

Besides, the occurrence of MCs in the oligochaete *Limnodilus hoffineisteri* and the insect larva *Chironomus* species as a source of possible exposure was investigated. The results from MCs analysis using LC-UV for detection in the studied larva suggested a potential transfer through the food web to other organisms such as carp [132]. Later, another group carried out a study to investigate the bioaccumulation of MCs in four different types of carp (silver carp, bighead carp, crucian carp, and common carp) collected from Lake Taihu [138]. The study concluded that the estimated daily intakes of MCs in 55.6% of the muscle samples exceeded the provisional TDI as high concentrations of MCs were detected in the analyzed muscle samples of these species (Table 3). Jiang et al. detected lower concentrations of MCs using ELISA in both silver carp and crucian carp collected from Lake Chaohu than those previously reported in shrimps and mussels by other researchers [51,132,139]. However, the same authors detected much higher MCs concentrations using the same technique for detection (ELISA) in a different study [140]. Very recently, 10 species of farmed fish, covering four feeding habits, were surveyed for the contamination of MC-LR and MC-RR, using LC-PDA and the results showed significantly higher concentrations of both MC-LR and MC-RR in silver carp and black carp than in other investigated species [141]. In a wider study, samples from 26 species of fish and shellfish were collected from three lakes (Taihu, Chaohu, and Dinachi) for MCs analyses. The estimated levels of human exposure to MCs in samples from lake Taihu, lake Chaohu, and lake Dinachi were, respectively, 5–184 times, 2–50 times, and 1.5–4 times higher than the provisional TDI [142]. As variations in MCs contamination among species and months were observed, the study recommended to adjust the legal fishing seasons or select certain species to harvest for the sake of safe seafood. The same recommendation was again highlighted in another recently published paper by the same author; however, the samples were collected in 2009–2010 [136]. By contrast, D. Zhang et al. reported that the consumption of silver carp fish from any lake of eight eutrophic Chinese lakes, located along the Yangtze River, is safe as the estimated daily intake (0.002 to 0.007 µg/kg day) was much lower the provisional TDI [143].

Total MCs in muscles of three edible fish species collected from Eğirdir, Turkey, ranged from 0.36 to 17.54 µg/kg dry weight using ELISA (Table 3) and had much higher levels after re-analyzing a few positive samples with LC-PDA. Also, the study revealed that MCs variants, but not MC-LR, are more concentrated in liver and muscle samples [144]. According to a previous report, CYN was reported for the first time in Turkey in 2013 [145]. Later, a survey to detect both MC-LR and CYN in different water bodies was conducted, however this has not been well investigated in Turkish seafood [146]. Two commercially cultivated fish species, carp (*Cyprinus carpio*) and catfish (*Clarias batrachus*), in Indian markets in Varanasi area had MCs at variable concentrations in the muscles, which the later species had 10-fold levels higher than carp, especially for the detected dominant variant MC-RR using LC-MS/MS for detection. According to the study, the results indicate a species specificity and variation in MCs adsorption and accumulation [147]. The only study for MC-LR contamination in Iranian fish (silver carp and northern pike) was conducted in the Anzali wetland [148]. Using LC-UV for detection, similar concentrations of MC-LR were found in muscles of both species which exceeded the permissible levels in case of human consumption. No significant variation in MC-LR concentrations in muscles among different seasons that were chosen for sampling. Two experimental studies were conducted in Thailand to study the accumulation of MCs in cultured Nile tilapia and freshwater prawns [149,150]. In both studies, higher levels of MC-LR were detected in prawn than in Nile tilapia fish which was above the provisional TDI. In these experiments, a water green system was used, however it is unclear whether this system as well as the feeding system make a significant contribution to MCs accumulation in the selected species [149,150].

In Vietnam, locally consumed bivalves (*Corbicula* and *Ensidens* sp.,) showed a high concentration of MCs, however, fishes from the same sampling area had undetectable levels of MCs in their muscles (Table 3) [151]. The author used LC-PDA to detect covalently bound MCs in both bivalves (390–780 µg/kg, DW) and fishes (60–90 µg/kg, DW). Another recent study from Vietnam, in which Apple snails (*Pomacea canaliculata*) were sampled and analyzed for eight MCs variants and NODs using LC-MS/MS. In the muscular part of Apple snails, MC-dmLR variant was detected in three samples. The authors also found 10 times higher concentrations of MC-dmLR in the viscera and, therefore, complete removal of the visceral part of these snails during food processing was advised. Other targeted MCs variants (dm-RR, MC-RR, MC-YR, dm-LR, MC-LR, MC-LY, MC-LW, MC-LF) and NODs were not detected in the analyzed *Mollusca* [152].

**Table 3:** Occurrence of cyanotoxins in seafood from the developing countries in Asia.

Country	Location/ Year	Detected Toxins and Concentration Ranges (μg/kg, DW or WW)	Matrix	N	P	Method of Detection	References
China	Tianjin/2017	MC-RR (up to 290, DW); MC-LR (up to120, DW)	*Carassius auratus* (muscles)	21	NM	LC-PDA	[153]
	Lake Taihu/2005	MC-RR (up to 2, DW)	*Hypophthalmichthys molitrix* (muscles)	9	NM	LC-MS/MS	[6]
	Lake Taihu/2004–2005	MCs (up to 887, DW)	*Aristichthys nobilis* (muscles)	48	NM	LC-MS/MS	[135]
	Lake Taihu/2003–2004	MCs (70.6–584, DW)	*Anodonta woodiana*; *Hyriopsis cumingii*; *Cristaria plicata*; *Lamprotula leai* (muscles)	28	15	LC-UV	[134]
	Lake Chaohu, Anhui Province/2003	MC-LR and -RR (up to 530, DW)	*Palae-monetes*; *Macrobrachium nipponensis* (shrimps)	11	5	LC-UV	[51]
	Lake Taihu/2004–2005	MCs (up to 1244, DW)	*Hypophthalmichthys molitrix* (muscles)	12	11	LC-MS/MS	[133]
	Lake Chaohu, Anhui Province/2003	MC-LR and -RR (up to 180, DW)	*Corbicula fluminea* (muscles)	7	4	LC-UV	[132]
	China’s Tiesha River, Hangzhou/NM	MCs (up to 2860, WW)	*Hypophthalmichthys molitrix* (muscles)	3	3	LC-MS/MS	[154]
	Lake Taihu/2011	MCs (25.5–31.7, DW)	*Hypophthalmichthys* *molitrix*; *Aristichthys nobilis*; *Carassius carassius*; *Cyprinus carpio* (muscles)	46	NM	LC-MS/MS	[138]
	Lake Chaohu, Anhui Province/2012	MCs (4.3–27.9, DW)	*Hypophthalmichthys molitrix*; *Carassius auratus* (muscles)	NM	NM	ELISA	[139]
	Lake Chaohu, Anhui Province/2014	MCs (1.3–350, DW)	*Hypophthalmichthys* *molitrix*; *Carassius auratus* (muscles)	60	NM	ELISA	[140]
	WJD Reservoir, Guizhou/2016–2017	MC-LR (1.0–450,WW) and MC-RR (1.7–678, WW)	*C. idellus*; *P. pekinensis*; *H. molitrix*; *A. nobilis*; *C. carpio*; *C. auratus*; *T. tinca*; *M. piceus*; *L. japonicas*; *P. asotus* (muscles)	205	106 (MC-LR), 110 (MCs-RR)	LC-PDA	[141]
	Fish pond, Hangzhou/2009	MC-LR (240, DW)	*Aristichthys nobilis* (muscles)	1	1	LC-MS/MS	[155]
	Lake Taihu and Lake Chaohu/2009–2010	MCs (18.6–48.2, DW)	Nine fish species (muscles)	NM	NM	ELISA	[136]
	Eight different lakes in four provinces (Hubei, Hunan, Jiangsu and Anhui)/2008	MCs (14–36, DW)	*Hypophthalmichthys* *molitrix* (muscles)	24	24	LC-MS/MS	[143]
	Lake Dianchi/2008	MCs (up to 1360, DW)	*Margarya melanioides* (whole snail)	6	6	ELISA	[156]
	Lake Chaohu, Anhui Prov-ince/2003	MC-LR (up to 2290, DW)	*Hypophthalmichthys* *molitrix*; *Carassius auratus*; *Cyprinus carpio*; *Coilia ectenes* (muscles)	8	4	LC-UV	[157]
	Lake Taihu/2005	MCs (13–170, DW)	*Hypophthalmichthys* *molitrix*; *Cyprinus carpio*; *Carassius auratus* (muscles)	NM	NM	LC-MS/MS	[137]
India	Lakshmikund pond, Varanasi/2011-2012	MC-LR (1.44–3.01, DW); MC-RR (5.94–65.03, DW); MC-YR (1.39–1.71, DW)	*Cyprinus carpio*; *Clarias batrachus* (muscles)	3	3	LC-MS/MS	[147]
Iran	Anzali wetland, Guilan Province/2014	MC-LR (10.1–40.9, WW)	*Hypophthalmichthys* *molitrix*; *Esox lucius* (muscles)	18	NM	LC-UV	[148]
Thailand	Fish ponds, Bang Bo District, Samut Prakan Province/NM	MC-LR < LOD	*Clarias* *macrocephalus* (muscles)	720	-	LC-MS/MS	[158]
	Phayao Lake, Phayao Province/2011	MC-LR (up to 0.26, DW)	*Oreochromis niloticus* (muscles)	24	7	LC-UV	[159]
	Experimental fish pound, Chiang Mai/NM	MC-LR (8.3–14.4, DW)	*Oreochromis niloticus* (muscles) and *Macrobrachium* *Rosenbergii* (whole prawns)	120	NM	ELISA	[149]
	Experimental fish pound, Chiang Mai/2006-2007	MC-LR (0.84 to 3.20, DW)	*Tilapia nilotica* (muscles) and *Macrobrachium* *Rosenbergii* (whole prawns)	30	NM	ELISA	[150]
Turkey	Lake Eğirdir, Turkey/2013	MCs (0.36–17.54, DW)	*Cyprinus carpio*; *Cyprinus gibelio*; *Atherina boyeri* (muscles)	110	51	ELISA	[144]
Vietnam	Mekong basin, South Vietnam/ 2015–2016	MC-dmLR (150–300, DW)	*Pomacea canaliculate* (muscles)	68	3	LC-MS/MS	[152]
	Phuoc Ninh/2011	MCs (1150–2370, DW)	*Corbicula* sp.; *Ensidens* sp. (whole bivalves)	200	200	LC-PDA	[151]

DW, dry weight; WW, wet or fresh weight; N, number of samples; P, positive samples; LOD, limit of detection; NM, not mentioned; MCs, microcystins; MC-LR, microcystin-LR; MC-RR, microcystin-RR; MC-YR, microcystin-YR; MC-dmLR, microcystin-dmLR; ELISA, enzyme-linked immunosorbent assay; LC-UV, liquid chromatography with ultraviolet detector; LC-PDA, liquid chromatography with photo-diode array detector; LC-MS/MS, liquid chromatography tandem mass spectrometry.

In humans, a cross-sectional study included 5493 individuals (southwest China) found an association between renal-function impairment and MC-LR exposure. This association was based on direct measurements of MC-LR in different sources of waters and types of seafoods using an ELISA assay together with questionnaires and determination of clinical renal function indicators [160]. The study noted low-dose of exposure to MC-LR over the long term. Recently, researchers have proposed an animal model to assess the risk of exposure to MC-LR present in drinking water over long periods [161]. However, to the best of our knowledge, no such model is available for cyanotoxins in seafood.

### 4.3. Occurrence of Cyanotoxins in Food from the Developing Countries in Latin American

In Latin America, data on cyanotoxin occurrence in (sea)food are available from Brazil followed by Mexico, Argentina, and Guatemala (Figure 4 and Table 4). The rest of the continent lacks information on this matter. These studies form 32.3% (21 out of 65 publications) of all the recognized publications from the developing countries in Africa, Asia, and Latin America. Five papers were excluded from the further discussion for the following reasons: one paper investigated the uptake of MCs in Yellow Clam in the lab in addition to a source of contamination reported by the authors; one paper showed the growth performance effect in *O. niloticus* fingerlings exposed to a saxitoxin-producing strain; and another three papers explored the uptake by the plants but under laboratory conditions. Therefore, 16 papers were presented in Table 4 including one paper focused on the occurrence of cyanotoxins in edible plants.

One of the most common fish species, *Geophagus brasiliensis*, in Brazil was analyzed for the presence of MCs, and it was found that the detected concentrations of MCs were below the recommended limits for human consumption (Table 4). The same authors studied the depuration of MCs in *G. brasiliensis* after 7, 15, 30, and 90 days of MCs exposure. The results showed that MCs can still be detected in muscles even after a period of three months [162]. Sliver carp (*Hypophthalmichthys molitrix*) consumers (Brasilia-FD, Brazil) would be exposed to a high level of MCs, especially in the dry season of winter, which is 240 times more than the recommended provisional TDI [163]. This was the main conclusion of a 1-year study on the seasonal dynamics of MCs bioaccumulation in silver carp tissues in Paranoa Lake in Brazil (Table 4). However, the authors did not indicate the contamination prevalence of the analyzed samples. Redbreast tilapia (*Tilapia rendalli*) in Rio de Janeiro, Brazil, bioaccumulate MCs in their muscles to levels that are 42 times above the recommended guidelines [54]. Nevertheless, the bioaccumulation of MCs in this species (both in the liver and muscle) seems to be lower than in Nile tilapia when both species were exposed to the same amount of MCs in a toxic bloom [164]. These results seem to be in line with another study conducted in Northeast Brazil in which the mortalities of Nile tilapia due to very high levels of MCs in liver samples (up to 2590 µg/kg) were recorded [165].

Other fish and crustaceans (carb and shrimps) from Sepetiba Bay in Brazil were analyzed with ELISA during an 11-month survey. In general, low levels of MCs were detected in all samples with a positive correlation with the MCs concentrations in seston samples and around 19% of fish muscle and crustacean samples were above the provisional TDI (0.04 µg/kg of body weight a day) set by the WHO [166]. Recently, Nile tilapia from Lake Zumpango, Mexico City, were found to have low levels of MCs using ELISA for detection, while the exposure level to the same toxins was estimated to be two times above the recommended levels in case of consumption of contaminated mesa silverside (*Chirostoma jordani*), which, according to the authors, is more locally consumed than tilapia fish [55]. This is in contrast to an earlier study examined the contamination of MCs in uncooked, cooked, and dried *Chirostoma* sp. collected from local markets in the state of Michoacan as MCs were not detected when they were analyzed with ELISA [167]. According to Cazenave et al. approximately 21% of the detected MC-RR using LC-UV in wild pejerrey fish (*Odontesthes bonariensis*) that are chronically exposed to toxic blooms in Cordoba, Argentina, is accumulated in the muscles (up to 340 µg/kg, WW) [168]. This might give an average of exposure equals to 5 µg MC-RR per day. Interestingly, Ame et al. found MCs in *O. bonariensis* fish muscles from Argentine, at low levels, in both wet and dry season. However, liver samples collected from the dry season seemed to be, according to the analytical method used, were not contaminated with MCs. As a result, the study suggested a different accumulation profile for MCs in this species [169]. This was in agreement with the previous study of Cazenave et al., as they detected higher MCs during the wet season [168].

Locally consumed tegogolo snails (*Pomacea patula catemacensis*) in Mexico had CYN and STXs at low concentrations after an ELISA analysis (Table 4), however, no toxins were detected in the same samples using LC-MS/MS. The authors discussed the results and justified this due to the analytical limitations in extraction and/or methods of detection they used for analysis [94]. In another later work, the same authors surveyed numerous edible fish species from the same area for both CYN and STXs. The results showed the occurrences of CYN and STXs at low levels (Table 4) [81]. On the other hand, Galvão et al. proposed a depuration period of five days for the STXs accumulated in Nile tilapia muscles to reach undetectable levels [93]. The toxic effects of STXs on the survival and growth performance of Nile tilapia fingerlings were shown to be dependent on their weight (more mortality in the exogenous feeding phase) [170]. Samples of *Geophagus brasiliensis* fish from the Alagados reservoir were all contaminated with PST (gonyautoxins) with similar levels in all sampling seasons (spring 2007, summer, and autumn 2008), and these levels (in STX eq./100 g) were safe in all the positive samples [95]. The only available study on NODs accumulation in seafood was conducted on shrimp (*Palaemonetes argentines*) samples collected from San Roque Reservoir, Argentina. The toxin was detected up to three weeks after exposure (0.16 ± 0.03 μg/g) using LC-MS/MS, making this species a potential carrier [66].

Bioaccumulation of MCs in edible plants and survey studies were conducted in Latin America in the last decade [24,25,58]. Two agricultural crops, tomato (*Solanum lycopersicum*) and pepper (*Capsicum annuum*), cultivated in two agricultural farms and irrigated with water from Lake Amatitlán, Guatemala, showed the ability to bioaccumulate MC-RR in fruits and seeds [24]. Similarly, Bittencourt-Oliveira et al. studied the bioaccumulation of MC-LR and MC-RR in lettuce by irrigating the plant with MCs-contaminated water at different concentrations for a period of 15 days. The bioaccumulation factor of MC-LR appeared to be three times higher than MC-RR bioaccumulation which is further increasing with higher MCs exposure [25]. The bioaccumulation and depuration of CYN in lettuce and arugula has been also investigated [84]. However, no studies are available on the natural occurrence of this toxin in cultivated plants in Latin and/ or Central America.

**Table 4:** Occurrence of cyanotoxins in (sea)food from the developing countries in Latin America.

Country	Location/ Year	Detected Toxins and Concentration Ranges (μg/kg, DW or WW)	Matrix	N	P	Method of Detection	References
Argentina	Los Padres Lake/2007	MCs (1.0–8.0, WW)	*Odontesthes bonariensis* (muscles)	20	NM	LC-MS/MS	[169]
	San Roque reservoir/2004	MC-RR (4.8–340, WW)	*Odontesthes bonariensis* (muscles)	30	NM	LC-UV	[168]
	San Roque Reservoir/2011	NODs (80–90, WW)	*Palaemonetes argentines* (whole shrimp)	80	NM	LC-MS/MS	[66]
Brazil	Rio de Janeiro/2003	MCs (0.9–12, WW)	*Oreochromis niloticus*; *Tilapia rendalli* (muscles)	27	27	ELISA	[164]
	Jacarepaguá lagoon/2012	MCs (up to 4, WW)	*Oreochromis niloticus* (muscles)	17	10	ELISA	[171]
	Alagados Reservoir/2007–2008	GTXs (22.1–30.6, WW)	*Geophagus brasiliensis* (muscles)	64	64	LC-FLD	[95]
	Paranoa Lake/2006–2007	MC-LR (up to 3380, WW)	*Hypophthalmichthys molitrix* (muscles)	78	NM	LC-PDA	[163]
	Sepetiba Bay, Rio de Janeiro/1999	MCs (up to 103.3, WW)	Fish muscles; shrimp; crab	33	16	ELISA	[166]
	Rio de Janeiro/1996–1999	MCs (1.6–337.3, WW)	*Tilapia rendalli* (muscles)	144	108	ELISA	[54]
	Garça city/2005	STXs (up to 22, WW)	*Oreochromis niloticus* (muscles)	24	NM	LC-FLD	[93]
	Iraí Reservoir, Paraná State/2015	MCs (up to 7, WW)	*Geophagus brasiliensis* (muscles)	50	NM	LC-MS/MS	[162]
Guatemala	Lake Amatitlán cost/NM	MC-RR (up to 1.03 and 1.16, DW)	*Solanum lycopersicum* (Tomato); *Capsicum annuum* (peppers)	12	12	LC/MS/MS	[24]
Mexico	Lago Catemaco, Veracruz/NM	CYN (up to 3.35, WW); STXs (up to 1.04, WW)	*Pomacea patula catemacensi* (snails)	NM	NM	ELISA	[94]
	Lake Catemaco, Veracruz/2009	CYN (0.09–1.26, WW); STXs (0.03–0.71, WW)	*Rhamidia* sp.; *Oreochromis aureus; Vieja* sp.; *V. fenestrate*; *Heterandria* *Jonesii*; *Bramocharax cabelleroi*; *Cichlasoma urophtalmus*; *C. helleri*; *Dorosoma mexicana* (Fish tissue)	14	14	ELISA	[81]
	Michoacan/2008–2009	MCs (4.99–157, WW)	*Cyprinus carpio* sp.; *Goodea* sp. (Fish tissue)	NM	NM	ELISA	[167]
	Lake Zumpango, Mexico City/2016–2017	MCs (5–24, DW)	*Chirostoma jordan* (muscles)	30	30	ELISA	[55]

DW, dry weight; WW, wet or fresh weight; N, number of samples; P, positive samples; LOD, limit of detection; NM, not mentioned; MCs, microcystins; MC-RR, microcystin-RR; MC-LR, microcystin-LR; NODs, nodularins; CYN, cylindrospermopsin; STXs; saxitoxins; GTXs, guanitoxin; ELISA, enzyme-linked immunosorbent assay; LC-UV, liquid chromatography with ultraviolet detector; LC-PDA, LC-FLD, liquid chromatography with fluorescence detectors; liquid chromatography with photo-diode array detector; LC-MS/MS, liquid chromatography tandem mass spectrometry.

## 5. Regulation and Mitigation of Cyanotoxins

To protect the public from the adverse effects of cyanotoxins, WHO listed them as an emerging public health issue and established a provisional TDI for chronic exposure to MC-LR of 0.04 μg/kg body weight and a provisional guideline value of 1 μg/L in drinking water (cell-bound and extra-cellular toxins) in 1998 [172,173]. In 2020, new provisional guidelines were proposed by WHO for MC-LR, CYN, STXs and ANTX [174,175,176,177]. Although the provisional TDI did not change for MC-LR, the provisional guidelines have been adapted to better represent the health impact. In conclusion, it has been recommended to take into account the concentration of MCs present for properly assessing the risk. The 1 µg/L value for drinking water is still in use for long-term exposure, while a new provisional guideline of 12 µg/L has been proposed for short-term exposure. ANTX only received a provisional guideline value for short-term exposure for drinking water (30 µg/L) due to a lack of long-term toxicological data. On the other hand, CYN has received three provisional guideline values. The guidelines for short- and long-term exposure in drinking water have been set on 0.7 µg/L and 3 µg/L, respectively. STX has received provisional guidelines for drinking water at 3 µg/L. Since 2020, the EU has adapted the 1 µg/L provisional guideline value as a minimum for drinking water [178]. However, no regulations have been set for any other member of cyanotoxins, except MC-LR, in (sea)food. South Africa has set 1.0 µg/L as provisional guidelines value in drinking water for MC-LR. In Asia, both China and Turkey are following a similar provisional guidelines value in drinking water set by WHO [141,144]. In Latin America, only Argentina, Brazil, and Uruguay have adopted the same values from WHO for total MCs in drinking water while for the recreational waters still to be approved by the authorities [179]. Two other toxins, CYN and STXs, were also regulated in Brazil at 15 and 3 μg/L, respectively. Finally, the authorities in Cuba restrict the irrigation when the total number of phytoplanktonic cells is >100,000 cells/mL [27].

For mitigation of toxic bloom formation and occurrence of cyanotoxins, various strategies have been suggested. However, few studies have been conducted in developing countries. Management of a toxic bloom is not a straightforward task since it requires prevention, monitoring, treatment of the bloom in the affected water body. Furthermore, it should be taken into consideration that the selected treatment(s) is able to remove both the toxin-loaded cyanobacteria without causing lysis as well as the already liberated toxins in water. Consequently, the integration of more than one strategy will be more effective than relying on a single strategy. Up until now, studies with a potential to decontaminate cyanotoxins in (sea)food have been rare to non-existent, either due to the limited occurrence data and/or the complex matrix which may add another layer of complexity to conducting such research. Furthermore, the possible change in food quality may also be another obstacle. Therefore, the following sections give a brief description on different approaches that can effectively eradicate toxic cyanobacterial bloom formation as indirect way to minimize the bioaccumulation of cyanotoxins in fish.

### 5.1. Management of Nutrients

Management of nutrients (carbon, nitrogen, and phosphorus) is considered an important long-term approach to prevent the occurrence of a toxic bloom which is enhanced under eutrophication. Consequently, efforts were made to decrease the chance of eutrophication by lowering the content of nutrients released into surface water. This is primarily done through improving the process of wastewater treatment which is not widely implemented in developing countries. In addition, the rational use of fertilizer and manure in agriculture and reduction of the anthropogenic activities will eventually decrease the nutrient loads. Another way to control eutrophication is through the application of a special type of clay (e.g., lanthanum modified bentonite) that has affinity to bind to phosphorus, which is a critical key factor in eutrophication. However, the ability of these clays is hindered by competing oxyanions [180]. In addition, this technique is not always economically feasible. For cultivated fishes, a depuration strategy could be a safe approach to reduce the level of cyanotoxins [93].

### 5.2. Chemical and Physical Approaches

Using chemical algicides to destroy the bloom has been agreed to be an urgent intervention [181]. Despite the efficacy of this approach, several subsequent disadvantages make this option not the most appropriate one. First, these compounds or a mixture of compounds such as copper sulfate, the herbicide diuron, and chlorin cause cell lysis and the liberation of cyanotoxins in water, thus exacerbating water quality problems [3,182,183]. Secondly, their persistence in the environment can harm the ecosystem. A third disadvantage arises from the fact that a wide range of potential (novel) toxic effects may occur in other aquatic organisms. Low concentrations of hydrogen peroxide were suggested to have a less destructive effect on the environment with good efficacy against cyanobacteria [184]. However, the possibility of bloom reformation may occur at the same spot. The ozone gas showed high efficacy against *M. aeruginosa*, however, the cell lysis rate was high, resulting in the risk of toxin liberation [182,185].

Adsorbent materials have been widely applied in water to remove of trace contaminants, including extra-cellular cyanotoxins [186]. Examples for these adsorbents are activated carbon and iron-based adsorbents [187,188]. These two materials have been documented in the literature; however, the results were variable [185]. Efficacy of activated carbon depends on water quality, type of base material, dose, particle size, and, more importantly, the nature of the toxin. It was found that mesoporous activated carbon is more effective in the adsorption of MCs than micro- or macro-sized ones. This was concluded after the treatment of MC-LR contaminated water samples (5–20 μg/L) with commercial mesoporous activated carbon for 10 min. MC-LR concentrations decreased to a level lower than 1 μg/L [189]. Furthermore, wood-based activated carbon achieves the highest removal rate among various materials, especially against STXs and MC-LR [190,191]. Iron-based adsorbents such as magnetic bentonite material have been tested against MC-LR in river water samples, and the results showed good affinity for adsorption, especially at pH 2.1 [192]. Similarly, low pH with high ionic strength was found to be more convenient and gave better results with other iron-based adsorbents, such as iron oxides, against MC-LR [193]. Indeed further research is required to improve these approaches and also to be tested against other cyanotoxins.

The application of germicidal UV at a wave length of 254 nm causes limited photolysis of the extra-cellular cyanotoxins, especially MC-LR, in water, by generate highly reactive hydroxyl radicals [194]. However, UV light irradiation followed by ozone had a better degradation rate for MC-LR and ANTX in water because more highly reactive hydroxyl radicals are produced [195,196,197]. The same was also observed with hydrogen peroxide [194,198]. Recently, Chintalapati and Mohseni achieved more than 90% degradation of MC-LR present in complex natural water using a combination of UV radiation at 254 nm and 185 nm, with the latter wavelength inducing advanced oxidation [199]. Still more studies would be useful to confirm the efficacy of these approaches against other cyanotoxins and their applicability in full-scale operations. Other light-driven oxidation technologies for MC-LR degradation in water have been reviewed elsewhere [200].

### 5.3. Biological Approach

Biological control is considered an environmentally safe approach as no chemicals are added, which therefore avoids the risk of having unknown metabolites of potential harmful effects on the environment and humans. However, it is not applied as much as the other chemical and physical approaches in cyanotoxin removal. Previous studies suggested the use of certain fish species such as silver carp and bighead carp to decrease the formation of bloom by grazing it [6,133,135]; however, the precise effect of cyanotoxin bacteria and their toxins on these fishes is not well known. Other studies focused on the bacterial degradation of cyanotoxins, especially MC-LR, using various species such as *Bacillus* sp., *Sphingomonas* sp., *Sphingopyxis* sp., *Bordetella* sp., *Lactobacillus rhamnosus* sp., *Pseudomonas* sp., *Rahnella aquatilis* sp., and *Rhodococcus* sp. [45,201]. Of these species, some strains could have high degradation rate of MC-LR and MC-RR, which may show a significant role in the natural biodegradation and removal of MCs from water [202,203]. Furthermore, a cocktail of bacterial strains isolated from a water reservoir was tested to biodegrade MCs, which has led to complete degradation of MC-LR [201]. Regardless of whether the biodegradation of cyanotoxins is aerobic or anaerobic, there are some critical factors (such as water temperature, pH, toxin type, concentration, exogenous nutrients, and biotic factor) that affect the efficacy of the process [204]. Doubtless to say, the biodegradation pathway is quite different among cyanotoxins, therefore, more research is needed to optimize the ability of these selected strains to biodegrade other MCs variants as well as other cyanotoxins.

### 5.4. Public Awareness

Awareness of the public can significantly reduce human exposure to cyanotoxins either due to recreational activities, drinking untreated water or consuming seafood. Furthermore, sound social practices and avoiding throwing organic/inorganic waste materials into water sources is a worthy approach to limit the danger of bacterial bloom formation. Highlighting the effect of climate change on the safety of consumed food in the media could be adopted under national funded programs in developing countries. Whether only awareness should be applied or restrictions on recreational activities or prohibition of any water-related activity, this depends on the detected levels of the monitored toxin. Doubtless, the presence of a categorical classification (low, medium, and high) of risk to cyanotoxins will be helpful in taking the appropriate action. However, toxin analysis must be preceded by phytoplankton counts and species identification to decide if chemical analysis of the toxins is required. On the other hand, governments should issue laws to ensure that undesirable industrial effluents are appropriately sanitized before reaching water bodies.

## 6. Conclusions and Outlook

Despite the great success achieved by the scientific community in many aspects regarding the production, analysis, bioaccumulation, biodegradation, and toxic effects of cyanobacterial toxins, more research should be conducted in the future to fill in the knowledge gap we still encounter in cyanotoxin research. On the basis of the current work, assessment of cyanotoxins in foods from developing countries still requires more investigation. This may include development and validation of more analytical methods for the simultaneous quantitation of multiple categories of cyanotoxins and their variants in different food matrices from developing countries [99,108,109,110,111,112]. Furthermore, more survey studies are needed to explore the occurrence of cyanotoxins in different types of seafood and edible plants, especially from the countries that have no data. In addition, the effect of cooking and food processing on MCs concentrations and other toxins need to be investigated, and whether the potential products from the degradation are less or more toxic than the parent compounds [205,206,207].

For realistic risk assessment, identification of biomarkers of exposure is needed to carry out appropriate biomonitoring studies in the exposed populations [208]. Recently, studies from developing countries have been published, showing the great value of biomonitoring of cyanotoxins as a direct approach for exposure assessment [209,210].

Since cyanotoxin-producing bacteria can be present in aquatic, terrestrial, and aerial environments alongside a wide range of other environmental and food contaminants, characterization of the mechanisms of action of cyanotoxins to estimate the possibility of any additive or synergistic or antagonist effects on interactions with other toxins and hazardous chemicals (i.e., the combined effects of a mixture of cyanotoxins and other chemicals) should be considered [211,212]. Zhang et al. showed that corn and durum wheat are among the most susceptible crops to MCs causing phytotoxic effects [26]. On the other hand, it is known that some mycotoxins such as deoxynivalenol and zearalenone, produced by certain toxigenic fungal species, are also phytotoxins posing a profound public health threat. A synergistic effect of MC-LR with two of the most common mycotoxins in food, aflatoxin B1 and fumonisin B1, was found with a significant cytotoxic effect in binary and ternary toxin mixtures in cell cultures [213]. Cook et al. showed that co-exposure to ANXTs and the organophosphorus compound led to an increased toxicity for ANTXs in the mouse brain [70]. Recently, the co-exposure effect of CYN and bisphenol A was revealed in the HepG2 cell line, and an antagonistic effect was concluded due to less DNA double-strand breaks [85].

In coming decades, due to global warming and other anthropogenic activities, it is expected that the frequency and magnitude of cyanobacterial blooms will increase in surface waters. Therefore, future research will have to take into consideration the effect of climate change on toxic bloom formation [9]. Developing meaningful models to predict bloom formation and toxin production as well as novel programs for monitoring in the environment will be a must to avoid unexpected appearance of cyanotoxins. The development of molecular and chemical in situ tools should perhaps be a priority for constant and real-time monitoring of toxic cyanobacteria species and screening of their toxins, respectively.

A last consideration is to pay more attention to improving building capacity in developing countries, as the shortage of advanced research instruments in addition to the insufficient laboratory experience of young researchers represents a major challenge. Therefore, calls for building capacity proposals by national and international funding agencies are needed. Such projects should include long-term goals to: (1) realize “human” capacity building, (2) conduct cutting-edge research directly related to the developing countries, (3) transfer and share knowledge, and (4) co-implement effective management approaches to mitigate cyanotoxins.

## Figures and Tables

**Table 1:** Advantages and limitations for biological and chemical methods used/developed for the detection of cyanotoxins.

Evaluation Parameters	Biological Methods			Chemical Methods			
	Mouse Bioassay	ELISA Assay	PPI Assay	LC-UV, LC-PDA, LC-FLD	LC-MS/MS	LC-HRMS	Sensors
Sensitive	low	✓	✓	✓	✓✓	✓✓✓	✓✓
Expensive	cheap	✓	✓	✓	✓✓	✓✓✓	cheap
Distinguished variants				✓	✓✓	✓✓✓	
Multiple classes of toxins				✓	✓✓	✓✓	✓
Quantitation		✓	✓	✓	✓	✓	✓
Retrospective analysis						✓	
In situ analysis							✓
Trendy					✓	✓	✓
Other	Ethical approval	Matrix interference	Not for all cyanotoxins	Derivatization for LC-FLD analysis	Highly trained analysts	Highly trained analysts	

ELISA, enzyme-linked immunosorbent assay; PPI, protein phosphatase inhibition assays; LC-UV, liquid chromatography with ultraviolet detector; LC-PDA, liquid chromatography with photodiode array detector; LC-FLD, liquid chromatography with fluorescence detectors; LC-MS/MS, liquid chromatography tandem mass spectrometry; LC-HRMS, liquid chromatography high-resolution mass spectrometry.

## Data Availability

Not applicable.

## Supplementary Materials

The following are available online at www.mdpi.com/article/10.3390/toxins13110786/s1, Figure S1: Number of articles published in each year between 2000 and October 2021 on the occurrence of cyanotoxins in different sources from developing countries in Africa, Table S1: Number of publications focused on the natural occurrence of cyanotoxins in seafood as well as different environmental and water samples from African developing countries between 2000 and October 2021, Figure S2: Number of articles published in each year between 2000 and October 2021 on the occurrence of cyanotoxins in different sources from the developing countries in Asia, Table S2: Number of publications focused on the natural occurrence of cyanotoxins in seafood as well as different environmental and water samples from Asian developing countries between 2000 and October 2021, Figure S3: Number of articles published in each year between 2000 and October 2021 on the occurrence of cyanotoxins in different sources from developing countries in Latin America, Table S3: Number of publications focused on the natural occurrence of cyanotoxins in seafood as well as different environmental and water samples from Latin American developing countries between 2000 and October 2021.
