# Supplementary material for: Cyanotoxins and Food Contamination in Developing Countries: Review of Their Types, Toxicity, Analysis, Occurrence and Mitigation Strategies
Source: Toxins (Basel). 2021 Nov 6;13(11):786. doi: 10.3390/toxins13110786 (PMC8619289; doi:10.3390/toxins13110786)
Supplement: Supplementary file 1 [file toxins-13-00786-s001.zip › FigureS1_Table_S1_Africa.pdf]

Supplementary Materials: Cyanotoxins and Food Contamination in Developing Countries: Review of Their Types, Toxicity, Analysis, Occurrence and Mitigation Strategies

Mohamed F. Abdallah, Wannes Van Hassel, Mirjana Andjelkovic, Annick Wilmotte and Andreja Rajkovic

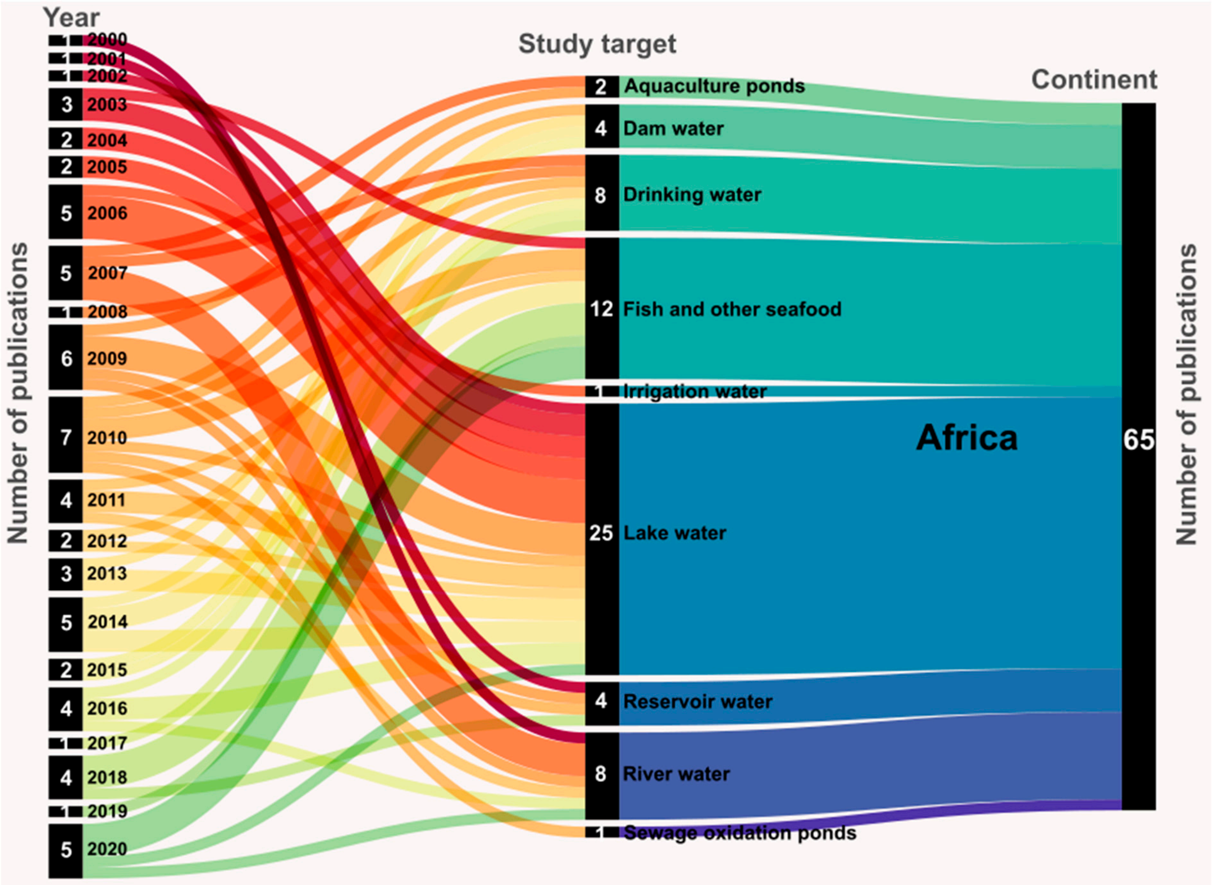

Figure S1. Number of articles published in each year between 2000 and October 2021 on the occurrence of cyanotoxins in different sources from the developing countries in Africa.

Table S1. Number of publications focused on the natural occurrence of cyanotoxins in seafood as well as different environmental and water samples from African developing countries between 2000 and 2021.

Country	Sample	Year	References
Algeria	Drinking water	2007	[1]
Algeria	Fish and other seafood	2014	[2]
Algeria	Lake water	2004	[3]
Algeria	Lake water	2016	[4]
Egypt	Aquaculture ponds	2007	[5]
Egypt	Drinking water	2015	[6]
Egypt	Drinking water	2016	[7]
Egypt	Fish and other seafood	2003	[8]
Egypt	Fish and other seafood	2018	[9]
Egypt	Fish and other seafood	2020	[10]
Egypt	Irrigation water	2006	[11]
Egypt	River water	2000	[12]
Egypt	River water	2007	[5]
Ethiopia	Fish and other seafood	2018	[13]
Ethiopia	Fish and other seafood	2020	[14]
Ethiopia	Lake water	2011	[15]
Ethiopia	Lake water	2014	[16]
Ethiopia	Reservoir water	2018	[17]
Ghana	Drinking water	2017	[18]
Kenya	Fish and other seafood	2018	[19]
Kenya	Fish and other seafood	2020	[20]
Kenya	Lake water	2003	[21]
Kenya	Lake water	2003	[22]
Kenya	Lake water	2004	[23]
Kenya	Lake water	2005	[24]
Kenya	Lake water	2006	[25]
Kenya	Lake water	2012	[26]
Kenya	Lake water	2013	[27]
Kenya	Sewage oxidation ponds	2010	[28]
Morocco	Lake water	2002	[29]
Morocco	Lake water	2017	[30]
Morocco	Reservoir water	2001	[31]
Morocco	Reservoir water	2009	[32]
Morocco	River water	2009	[33]
Mozambique	Dam water	2013	[34]
Mozambique	Drinking water	2012	[35]
Mozambique	Lake water	2011	[36]
Nigeria	Aquaculture ponds	2009	[37]
Nigeria	Lake water	2009	[38]
Nigeria	River water	2007	[39]
Nigeria	River water	2020	[40]
Senegal	Lake water	2006	[24]

South Africa	Dam water	2010	[41]
South Africa	Dam water	2014	[42]
South Africa	Dam water	2015	[43]
South Africa	Drinking water	2008	[44]
South Africa	Fish and other seafood	2014	[45]
South Africa	Fish and other seafood	2019	[46]
South Africa	Lake water	2009	[47]
South Africa	River water	2011	[48]
South Africa	River water	2016	[49]
Tanzania	Lake water	2005	[50]
Tanzania	Lake water	2006	[51]
Tanzania	Lake water	2013	[52]
Tanzania	Lake water	2014	[53]
Tanzania	Lake water	2020	[54]
Tunisia	Reservoir water	2010	[55]
Tunisia	River water	2007	[56]
Uganda	Fish and other seafood	2010	[57]
Uganda	Fish and other seafood	2010	[58]
Uganda	Fish and other seafood	2011	[59]
Uganda	Lake water	2009	[60]
Uganda	Lake water	2010	[61]
Zimbabwe	Drinking water	2010	[62]
Zimbabwe	Lake water	2006	[63]

References

1. Nasri: H.; Bouaïcha, N.; Harche, M.K. A new morphospecies of Microcystis sp. forming bloom in the cheffia dam (Algeria): Seasonal variation of microcystin concentrations in raw water and their removal in a full-scale treatment plant. *Environmental Toxicology* **2007**, *22*, 347–356, doi:10.1002/tox.20275.
2. Amrani, A.; Nasri, H.; Azzouz, A.; Kadi, Y.; Bouaiċha, N. Variation in cyanobacterial hepatotoxin (microcystin) content of water samples and two species of fishes collected from a shallow lake in Algeria. *Archives of Environmental Contamination and Toxicology* **2014**, *66*, 379–389, doi:10.1007/s00244-013-9993-2.
3. Nasri, A.B.; Bouaïcha, N.; Fastner, J. First Report of a Microcystin-Containing Bloom of the Cyanobacteria Microcystis spp. in Lake Oubeira, Eastern Algeria. *Archives of Environmental Contamination and Toxicology* **2004**, *46*, 197–202, doi:10.1007/s00244-003-2283-7.
4. Bouhaddada, R.; Nélieu, S.; Nasri, H.; Delarue, G.; Bouaïcha, N. High diversity of microcystins in a Microcystis bloom from an Algerian lake. *Environmental Pollution* **2016**, *216*, 836–844, doi:10.1016/j.envpol.2016.06.055.
5. Mohamed, Z.A. First report of toxic Cylindrospermopsis raciborskii and Raphidiopsis mediterranea (Cyanoprokaryota) in Egyptian fresh waters. *FEMS Microbiology Ecology* **2007**, *59*, 749–761, doi:10.1111/j.1574-6941.2006.00226.x.
6. Mohamed, Z.A.; Deyab, M.A.; Abou-Dobara, M.I.; El-Sayed, A.K.; El-Raghi, W.M. Occurrence of cyanobacteria and microcystin toxins in raw and treated waters of the Nile River, Egypt: implication for water treatment and human health. *Environmental Science and Pollution Research* **2015**, *22*, 11716–11727, doi:10.1007/s11356-015-4420-z.
7. Mohamed, Z.A.; Deyab, M.A.; Abou-Dobara, M.I.; El-Raghi, W.M. Occurrence of toxic cyanobacteria and microcystin toxin in domestic water storage reservoirs, Egypt. *Journal of Water Supply: Research and Technology - AQUA* **2016**, *65*, 431–440, doi:10.2166/aqua.2016.115.
8. Mohamed, Z.A.; Carmichael, W.W.; Hussein, A.A. Estimation of microcystins in the freshwater fish Oreochromis niloticus in an Egyptian fish farm containing a Microcystis bloom. *Environmental Toxicology* **2003**, *18*, 137–141, doi:10.1002/tox.10111.
9. Mohamed, Z.A.; Bakr, A. Concentrations of cylindrospermopsin toxin in water and tilapia fish of tropical

fishponds in Egypt, and assessing their potential risk to human health. *Environmental Science and Pollution Research* **2018**, *25*, 36287–36297, doi:10.1007/s11356-018-3581-y.
10. Mohamed, Z.; Ahmed, Z.; Bakr, A.; Hashem, M.; Alamri, S. Detection of free and bound microcystins in tilapia fish from Egyptian fishpond farms and its related public health risk assessment. *Journal fur Verbraucherschutz und Lebensmittelsicherheit* **2020**, *15*, 37–47, doi:10.1007/s00003-019-01254-0.
11. Mohamed, Z.A.; El-Sharouny, H.M.; Ali, W.S.M. Microcystin production in benthic mats of cyanobacteria in the Nile River and irrigation canals, Egypt. *Toxicon* **2006**, *47*, 584–590, doi:10.1016/j.toxicon.2006.01.029.
12. Brittain, S.; Mohamed, Z.A.; Wang, J.; Lehmann, V.K.B.; Carmichael, W.W.; Rinehart, K.L. Isolation and characterization of microcystins from a River Nile strain of Oscillatoria tenuis Agardh ex Gomont. *Toxicon* **2000**, *38*, 1759–1771, doi:10.1016/S0041-0101(00)00105-7.
13. Zewde, T.W.; Johansen, J.A.; Kifle, D.; Demissie, T.B.; Hansen, J.H.; Tadesse, Z. Concentrations of microcystins in the muscle and liver tissues of fish species from Koka reservoir, Ethiopia: A potential threat to public health. *Toxicon* **2018**, *153*, 85–95.
14. Zewde, T.W.; Kifle, D.; Johansen, J.A.; Demissie, T.B.; Hansen, J.H.; Tadesse, Z. Cyanobacterial abundance and microcystins in water, seston and fish tissues in Lake Hora-Arsedi (Ethiopia). *African Journal of Aquatic Science* **2020**, *45*, 475–485, doi:10.2989/16085914.2020.1723485.
15. Willén, E. Cyanotoxin production in seven Ethiopian Rift Valley Lakes. *Inland Waters* **2011**, *1*, 81–91, doi:10.5268/iw-1.2.391.
16. Mankiewicz-Boczek, J.; Gagala, I.; Jurczak, T.; Urbaniak, M.; Negussie, Y.Z.; Zalewski, M. Incidence of microcystin-producing cyanobacteria in Lake Tana, the largest waterbody in Ethiopia. *African Journal of Ecology* **2014**, *53*, 54–63, doi:10.1111/aje.12170.
17. Major, Y.; Kifle, D.; Spoof, L.; Meriluoto, J. Cyanobacteria and microcystins in Koka reservoir (Ethiopia). *Environmental Science and Pollution Research* **2018**, *25*, 26861–26873, doi:10.1007/s11356-018-2727-2.
18. Addico, G.N.D.; Hardege, J.D.; Kohoutek, J.; Degraft-Johnson, K.A.A.; Babica, P. Cyanobacteria and microcystin contamination in untreated and treated drinking water in Ghana. *Advances in Oceanography and Limnology* **2017**, *8*, doi:10.4081/aiol.2017.6323.
19. Simiyu, B.M.; Oduor, S.O.; Rohrlack, T.; Sitoki, L.; Kurmayer, R. Microcystin content in phytoplankton and in small fish from eutrophic nyanza gulf, lake Victoria, Kenya. *Toxins* **2018**, *10*, 275, doi:10.3390/toxins10070275.
20. Roegner, A.; Sitoki, L.; Weirich, C.; Corman, J.; Owage, D.; Umami, M.; Odada, E.; Miruka, J.; Ogari, Z.; Smith, W.; et al. Harmful Algal Blooms Threaten the Health of Peri-Urban Fisher Communities: A Case Study in Kisumu Bay, Lake Victoria, Kenya. *Exposure and Health* **2020**, *12*, 835–848, doi:10.1007/s12403-019-00342-8.
21. Ballot, A.; Pflugmacher, S.; Wiegand, C.; Kotut, K.; Krienitz, L. Cyanobacterial toxins in Lake Baringo, Kenya. *Limnologica* **2003**, *33*, 2–9, doi:10.1016/S0075-9511(03)80003-8.
22. Krienitz, L.; Ballot, A.; Kotut, K.; Wiegand, C.; PÃ¼tz, S.; Metcalf, J.S.; Codd, G.A.; Pflugmacher, S. Contribution of hot spring cyanobacteria to the mysterious deaths of Lesser Flamingos at Lake Bogoria, Kenya. *FEMS Microbiology Ecology* **2003**, *43*, 141–148, doi:10.1111/j.1574-6941.2003.tb01053.x.
23. Ballot, A.; Krienitz, L.; Kotut, K.; Wiegand, C.; Metcalf, J.S.; Codd, G.A.; Pflugmacher, S. Cyanobacteria and cyanobacterial toxins in three alkaline Rift Valley lakes of Kenya - Lakes Bogoria, Nakuru and Elmenteita. *Journal of Plankton Research* **2004**, *26*, 925–935, doi:10.1093/plankt/fbh084.
24. Ballot, A.; Krienitz, L.; Kotut, K.; Wiegand, C.; Pflugmacher, S. Cyanobacteria and cyanobacterial toxins in the alkaline crater lakes Sonachi and Simbi, Kenya. *Harmful Algae* **2005**, *4*, 139–150, doi:10.1016/j.hal.2004.01.001.
25. Kotut, K.; Ballot, A.; Krienitz, L. Toxic cyanobacteria and their toxins in standing waters of Kenya: Implications for water resource use. *Journal of Water and Health* **2006**, *4*, 233–245, doi:10.2166/wh.2006.0020.
26. Sitoki, L.; Kurmayer, R.; Rott, E. Spatial variation of phytoplankton composition, biovolume, and resulting microcystin concentrations in the Nyanza Gulf (Lake Victoria, Kenya). *Hydrobiologia* **2012**, *691*, 109–122, doi:10.1007/s10750-012-1062-8.
27. Krienitz, L.; Dadheech, P.K.; Fastner, J.; Kotut, K. The rise of potentially toxin producing cyanobacteria in Lake Naivasha, Great African Rift Valley, Kenya. *Harmful Algae* **2013**, *27*, 42–51, doi:10.1016/j.hal.2013.04.005.
28. Kotut, K.; Ballot, A.; Wiegand, C.; Krienitz, L. Toxic cyanobacteria at Nakuru sewage oxidation ponds - A potential threat to wildlife. *Limnologica* **2010**, *40*, 47–53, doi:10.1016/j.limno.2009.01.003.
29. Sabour, B.; Loudiki, M.; Oudra, B.; Vasconcelos, V.; Martins, R.; Oubraim, S.; Fawzi, B. Toxicology of a Microcystis ichthyoblabe waterbloom from Lake Oued Mellah (Morocco). *Environmental Toxicology* **2002**, *17*, 24–31, doi:10.1002/tox.10028.

30. Douma, M.; Ouahid, Y.; Loudiki, M.; del Campo, F.F.; Oudra, B. The first detection of potentially toxic Microcystis strains in two Middle Atlas Mountains natural lakes (Morocco). *Environmental Monitoring and Assessment* **2017**, *189*, doi:10.1007/s10661-016-5753-x.
31. Oudra, B.; Loudiki, M.; Sbiyyaa, B.; Martins, R.; Vasconcelos, V.; Namikoshi, N. Isolation, characterization and quantification of microcystins (heptapeptides hepatotoxins) in Microcystis aeruginosa dominated bloom of Lalla Takerkoust lake-reservoir (Morocco). *Toxicon* **2001**, *39*, 1375–1381, doi:10.1016/S0041-0101(01)00093-9.
32. Douma, M.; Ouahid, Y.; Campo, F.F.D.; Loudiki, M.; Mouhri, K.; Oudra, B. Identification and quantification of cyanobacterial toxins (microcystins) in two Moroccan drinking-water reservoirs (Mansour Eddahbi, Almassira). *Environmental Monitoring and Assessment* **2010**, *160*, 439–450, doi:10.1007/s10661-008-0708-5.
33. Oudra, B.; Dadi-El Andaloussi, M.; Vasconcelos, V.M. Identification and quantification of microcystins from a Nostoc muscorum bloom occurring in Oukaïmeden river (High-Atlas mountains of Marrakech, Morocco). *Environmental Monitoring and Assessment* **2009**, *149*, 437–444, doi:10.1007/s10661-008-0220-y.
34. Olivia, P.; Elisabeth, L.; Dacia, C.; Luis, N.; Janneche, U.S.; Morten, S.; vik; Knut, G.B. Quantification of microcystin-producing microcystis in freshwater bodies in the Southern Mozambique using quantitative real time polymerase chain reaction. *African Journal of Biotechnology* **2013**, *12*, 4850–4857, doi:10.5897/ajb12.2023.
35. Pedro, O.; Correia, D. Occurrence of microcystins in freshwater bodies in Southern Mozambique. *Journal of Research in Environmental Science and Toxicology* **2012**, *1*, 58–65.
36. Pedro, O.; Correia, D.; Lie, E.; Skåre, J.U.; Leão, J.; Neves, L.; Sandvik, M.; Berdal, K.G. Polymerase chain reaction (PCR) detection of the predominant microcystin-producing genotype of cyanobacteria in Mozambican lakes. *African Journal of Biotechnology* **2011**, *10*, 19299–19308, doi:10.5897/AJB11.1521.
37. Chia, A.M.; Abolude, D.S.; Ladan, Z.; Akanbi, O.; Kalaboms, A. The presence of microcystins in aquatic ecosystems in Northern Nigeria: Zaria as a case study. *Research Journal of Environmental Toxicology* **2009**, *3*, 170–178, doi:10.3923/rjet.2009.170.178.
38. Chia, A.M.; Oniye, S.J.; Ladan, Z.; Lado, Z.; Pila, A.E.; Inekwe, V.U.; Mmerole, J.U. A survey for the presence of microcystins in aquaculture ponds in Zaria, Northern-Nigeria: Possible public health implication. *African Journal of Biotechnology* **2009**, *8*, 6282–6289, doi:10.5897/AJB09.1263.
39. Odokuma, L.O.; Isirima, J.C. Distribution of cyanotoxins in aquatic environments in the Niger Delta. *African Journal of Biotechnology* **2007**, *6*, 2375–2385, doi:10.5897/ajb2007.000-2373.
40. Kadiri, M.O.; Isagba, S.; Ogbebor, J.U.; Omoruyi, O.A.; Unusiotame-Owolagba, T.E.; Lorenzi, A.S.; Bittencourt-Oliveira, M. do C.; Chia, M.A. The presence of microcystins in the coastal waters of Nigeria, from the Bights of Bonny and Benin, Gulf of Guinea. *Environmental Science and Pollution Research* **2020**, *27*, 35284–35293, doi:10.1007/s11356-020-09740-x.
41. Masango, M.G.; Myburgh, J.G.; Labuschagne, L.; Govender, D.; Bengis, R.G.; Naicker, D. Assessment of microcystis bloom toxicity associated with wildlife mortality in the kruger national park, South Africa. *Journal of Wildlife Diseases* **2010**, *46*, 95–102, doi:10.7589/0090-3558-46.1.95.
42. Ballot, A.; Sandvik, M.; Rundberget, T.; Botha, C.J.; Miles, C.O. Diversity of cyanobacteria and cyanotoxins in Hartbeespoort Dam, South Africa. *Marine and Freshwater Research* **2014**, *65*, 175–189, doi:10.1071/MF13153.
43. Oberholster, P.J.; Jappie, S.; Cheng, P.H.; Botha, A.M.; Matthews, M.W. First report of an Anabaena Bory strain containing microcystin-LR in a freshwater body in Africa. *African Journal of Aquatic Science* **2015**, *40*, 21–36, doi:10.2989/16085914.2014.993583.
44. Fosso-Kankeu, E.; Jagals, P.; Du Preez, H. Exposure of rural households to toxic cyanobacteria in container-stored water. *Water SA* **2008**, *34*, 631–636, doi:10.4314/wsa.v34i5.180660.
45. Nchabeleng, T.; Cheng, P.; Oberholster, P.J.; Botha, A.M.; Smit, W.J.; Luus-Powell, W.J. Microcystin-LR equivalent concentrations in fish tissue during a postbloom Microcystis exposure in Loskop Dam, South Africa. *African Journal of Aquatic Science* **2014**, *39*, 459–466, doi:10.2989/16085914.2014.973830.
46. Modley, L.A.S.; Rampedi, I.T.; Avenant-Oldewage, A.; Mhuka, V.; Nindi, M.; Van Dyk, C. Microcystin concentrations and liver histopathology in Clarias gariepinus and Oreochromis mossambicus from three impacted rivers flowing into a hyper-eutrophic freshwater system: A pilot study. *Environmental Toxicology and Pharmacology* **2019**, *71*, 103222, doi:10.1016/j.etap.2019.103222.
47. Oberholster, P.J.; Myburgh, J.G.; Govender, D.; Bengis, R.; Botha, A.M. Identification of toxigenic Microcystis strains after incidents of wild animal mortalities in the Kruger National Park, South Africa. *Ecotoxicology and Environmental Safety* **2009**, *72*, 1177–1182, doi:10.1016/j.ecoenv.2008.12.014.
48. Makhera, M.; Gumbo, J.R.; Chigayo, K. Monitoring of microcystin-LR in Luvuvhu River catchment: Implications for human health. *African Journal of Biotechnology* **2011**, *10*, 405–412, doi:10.5897/AJB10.1280.
49. Eguzozie, K.; Mavumengwana, V.; Nkosi, D.; Kayitesi, E.; Nnabuo-Eguzozie, E.C. Bioaccumulation and

Quantitative Variations of Microcystins in the Swartspruit River, South Africa. *Archives of Environmental Contamination and Toxicology* **2016**, *71*, 286–296, doi:10.1007/s00244-016-0269-5.
50. Sekadende, B.C.; Lyimo, T.J.; Kurmayer, R. Microcystin production by cyanobacteria in the Mwanza Gulf (Lake Victoria, Tanzania). *Hydrobiologia* **2005**, *543*, 299–304, doi:10.1007/s10750-004-6949-6.
51. Lugomela, C.; Pratap, H.B.; Mgaya, Y.D. Cyanobacteria blooms-A possible cause of mass mortality of Lesser Flamingos in Lake Manyara and Lake Big Momela, Tanzania. *Harmful Algae* **2006**, *5*, 534–541, doi:10.1016/j.hal.2005.10.001.
52. Miles, C.O.; Sandvik, M.; Nonga, H.E.; Rundberget, T.; Wilkins, A.L.; Rise, F.; Ballot, A. Identification of microcystins in a Lake Victoria cyanobacterial bloom using LC-MS with thiol derivatization. *Toxicon* **2013**, *70*, 21–31, doi:10.1016/j.toxicon.2013.03.016.
53. Cornelissen, I.J.M.; Silsbe, G.M.; Verreth, J.A.J.; van Donk, E.; Nagelkerke, L.A.J. Dynamics and limitations of phytoplankton biomass along a gradient in Mwanza Gulf, southern Lake Victoria (Tanzania). *Freshwater Biology* **2014**, *59*, 127–141, doi:10.1111/fwb.12253.
54. Mchau, G.J.; Machunda, R.; Kimanya, M.; Makule, E.; Gong, Y.Y.; Mpolya, E.; Meneely, J.P.; Elliott, C.T.; Greer, B. First Report of the Co-occurrence of Cylindrospermopsin, Nodularin and Microcystins in the Freshwaters of Lake Victoria, Tanzania. *Exposure and Health* **2020**, 1–10, doi:10.1007/s12403-020-00372-7.
55. Fathalli, A.; Ben Rejeb Jenhani, A.; Moreira, C.; Welker, M.; Romdhane, M.; Antunes, A.; Vasconcelos, V. Molecular and phylogenetic characterization of potentially toxic cyanobacteria in Tunisian freshwaters. *Systematic and Applied Microbiology* **2011**, *34*, 303–310, doi:10.1016/j.syapm.2010.12.003.
56. El Herry, S.; Bouaïcha, N.; Jenhani-Ben, R.A.; Romdhane, M.S. First observation of microcystins in tunisian inland waters: A threat to river mouths and lagoon ecosystems. *Transitional Waters Bulletin* **2007**, *1*, 73–82, doi:10.1285/i1825229Xv1n2p73.
57. Nyakairu, G.W.A.; Nagawa, C.B.; Mbabazi, J. Assessment of cyanobacteria toxins in freshwater fish: A case study of Murchison Bay (Lake Victoria) and Lake Mburo, Uganda. *Toxicon* **2010**, *55*, 939–946, doi:10.1016/j.toxicon.2009.07.024.
58. Semyalo, R.; Rohrlack, T.; Naggawa, C.; Nyakairu, G.W. Microcystin concentrations in Nile tilapia (Oreochromis niloticus) caught from Murchison Bay, Lake Victoria and Lake Mburo: Uganda. *Hydrobiologia* **2010**, *638*, 235–244, doi:10.1007/s10750-009-0047-8.
59. Poste, A.E.; Hecky, R.E.; Guildford, S.J. Evaluating microcystin exposure risk through fish consumption. *Environmental Science and Technology* **2011**, *45*, 5806–5811, doi:10.1021/es200285c.
60. Okello, W.; Portmann, C.; Erhard, M.; Gademann, K.; Kurmayer, R. Occurrence of microcystin-producing cyanobacteria in Ugandan freshwater habitats. *Environmental Toxicology* **2010**, *25*, 367–380, doi:10.1002/tox.20522.
61. Okello, W.; Ostermaier, V.; Portmann, C.; Gademann, K.; Kurmayer, R. Spatial isolation favours the divergence in microcystin net production by Microcystis in Ugandan freshwater lakes. *Water Research* **2010**, *44*, 2803–2814, doi:10.1016/j.watres.2010.02.018.
62. Mhlanga, L.; Day, J.; Cronberg, G.; Chimbari, M.; Siziba, N.; Annadotter, H. Cyanobacteria and cyanotoxins in the source water from Lake Chivero, Harare, Zimbabwe, and the presence of cyanotoxins in drinking water. *African Journal of Aquatic Science* **2006**, *31*, 165–173, doi:10.2989/16085910609503888.
63. Ndebele, M.R.; Magadza, C.H.D. The occurrence of microcystin-LR in Lake Chivero, Zimbabwe. *Lakes and Reservoirs: Research and Management* **2006**, *11*, 57–62, doi:10.1111/j.1440-1770.2006.00287.x.
