# Supplementary material for: Cyanotoxins and Food Contamination in Developing Countries: Review of Their Types, Toxicity, Analysis, Occurrence and Mitigation Strategies
Source: Toxins (Basel). 2021 Nov 6;13(11):786. doi: 10.3390/toxins13110786 (PMC8619289; doi:10.3390/toxins13110786)
Supplement: Supplementary file 1 [file toxins-13-00786-s001.zip › FigureS2_Table_S2_Asia.pdf]

Supplementary Materials: Cyanotoxins and Food Contamination in Developing Countries: Review of Their Types, Toxicity, Analysis, Occurrence and Mitigation Strategies

Mohamed F. Abdallah, Wannes Van Hassel, Mirjana Andjelkovic, Annick Wilmotte and Andreja Rajkovic

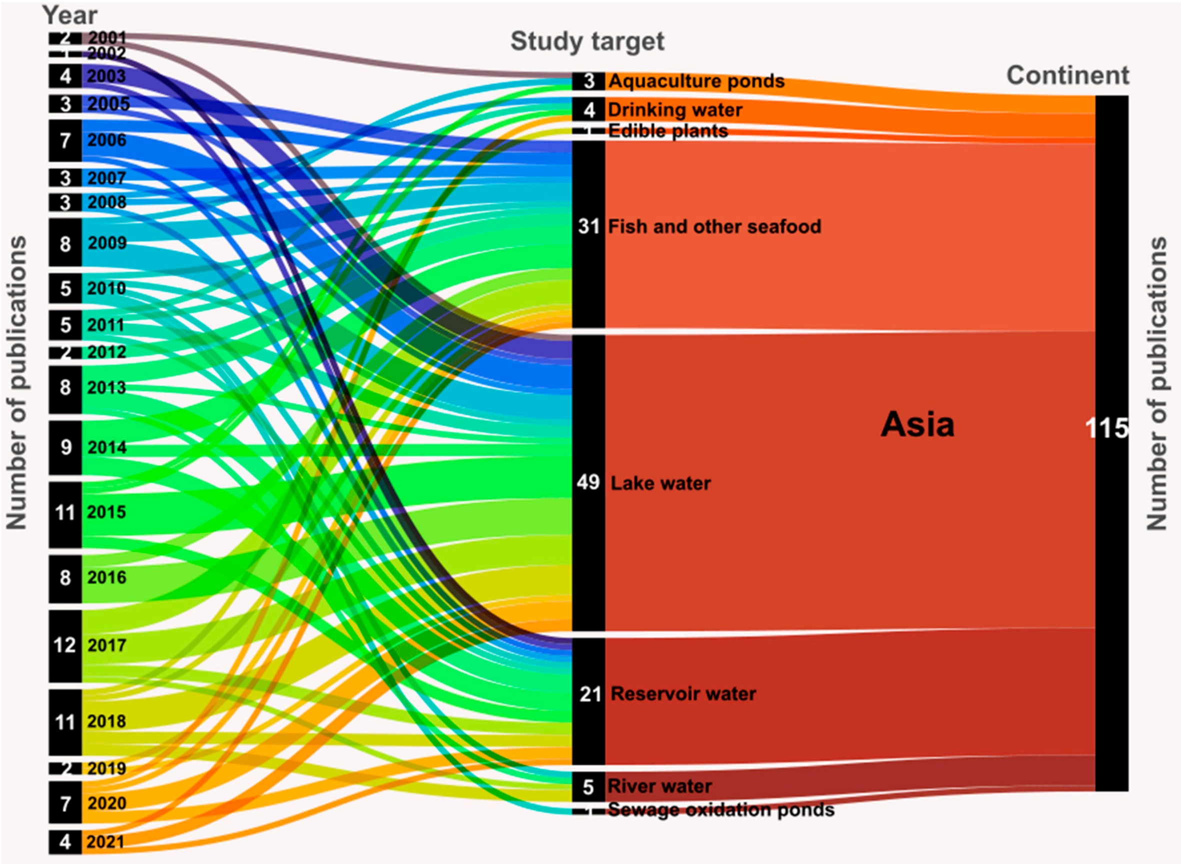

Figure S2. Number of articles published in each year between 2000 until October 2021 on cyanotoxin occurrence in different sources from the developing countries in Asia.

Table S2. Number of publications focused on the natural occurrence of cyanotoxins in seafood as well as different environmental and water samples from Asian developing countries between 2000 and 2021.

Country	Sample	Year	References
Armenia	Lake water	2020	[1]
Armenia	Lake water	2018	[2]
Bangladesh	Drinking water	2015	[3]
Bangladesh	Aquaculture ponds	2009	[4]
Bangladesh	Sewage oxidation ponds	2010	[5]
China	Fish and other seafood	2019	[6]
China	Edible plants	2018	[7]
China	Fish and other seafood	2008	[8]
China	Fish and other seafood	2005	[9]
China	Fish and other seafood	2005	[10]
China	Fish and other seafood	2006	[11]
China	Fish and other seafood	2007	[12]
China	Fish and other seafood	2009	[13]
China	Reservoir water	2008	[14]
China	Lake water	2016	[15]
China	River water	2018	[16]
China	Fish and other seafood	2014	[17]
China	Fish and other seafood	2014	[18]
China	Fish and other seafood	2017	[19]
China	Fish and other seafood	2021	[20]
China	Reservoir water	2014	[21]
China	Lake water	2007	[22]
China	Lake water	2016	[23]
China	Lake water	2017	[24]
China	Fish and other seafood	2017	[25]
China	Fish and other seafood	2010	[26]
China	Fish and other seafood	2020	[27]
China	Fish and other seafood	2012	[28]
China	Lake water	2013	[29]
China	Lake water	2015	[30]
China	Lake water	2003	[31]
China	Lake water	2015	[32]
China	Lake water	2018	[33]
China	River water	2013	[34]
China	Lake water	2020	[35]
China	Lake water	2010	[36]
China	Lake water	2006	[37]
China	River water	2018	[38]
China	Fish and other seafood	2005	[39]
China	Reservoir water	2011	[40]
China	Lake water	2016	[41]
China	Lake water	2018	[42]
China	Lake water	2020	[43]
China	Lake water	2006	[44]
China	Lake water	2016	[45]
China	Drinking water	2011	[46]
China	Lake water	2014	[47]

China	Fish and other seafood	2007	[48]
China	Drinking water	2008	[49]
China	Fish and other seafood	2009	[50]
China	Fish and other seafood	2009	[51]
China	River water	2010	[52]
China	Fish and other seafood	2012	[53]
China	Fish and other seafood	2013	[54]
China	Lake water	2015	[55]
China	Lake water	2018	[56]
China	Fish and other seafood	2006	[57]
China	Lake water	2021	[58]
India	Lake water	2015	[59]
India	River water	2017	[60]
India	Lake water	2006	[61]
India	Lake water	2009	[62]
India	Lake water	2009	[63]
India	Fish and other seafood	2014	[64]
India	Aquaculture ponds	2015	[65]
Iran	Fish and other seafood	2017	[66]
Philippines	Lake water	2003	[67]
Russia	Lake water	2011	[68]
Russia	Lake water	2011	[69]
Russia	Lake water	2015	[70]
Russia	Reservoir water	2015	[71]
Russia	Lake water	2016	[72]
Russia	Lake water	2016	[73]
Russia	Lake water	2017	[74]
Russia	Reservoir water	2020	[75]
Russia	Reservoir water	2018	[76]
Russia	Lake water	2015	[77]
Russia	Drinking water	2020	[78]
Russia	Reservoir water	2013	[79]
Russia	Reservoir water	2015	[80]
Sri Lanka	Reservoir water	2006	[81]
Thailand	Aquaculture ponds	2001	[82]
Thailand	Fish and other seafood	2011	[83]
Thailand	Fish and other seafood	2013	[84]
Thailand	Fish and other seafood	2014	[85]
Thailand	Lake water	2006	[86]
Thailand	Reservoir water	2013	[87]
Thailand	Reservoir water	2002	[88]
Thailand	Fish and other seafood	2013	[89]
Turkey	Lake water	2009	[90]
Turkey	Reservoir water	2014	[91]
Turkey	Lake water	2014	[92]
Turkey	Lake water	2017	[93]
Turkey	Reservoir water	2003	[94]
Turkey	Lake water	2003	[95]
Turkey	Lake water	2005	[96]
Turkey	Lake water	2009	[97]
Turkey	Fish and other seafood	2016	[98]

Turkey	Lake water	2015	[99]
Turkey	Lake water	2021	[100]
Turkey	Lake water	2017	[101]
Turkey	Reservoir water	2017	[102]
Turkey	Lake water	2017	[103]
Turkey	Lake water	2018	[104]
Turkey	Lake water	2019	[105]
Vietnam	Reservoir water	2010	[106]
Vietnam	Reservoir water	2013	[107]
Vietnam	Reservoir water	2014	[108]
Vietnam	Lake water	2001	[109]
Vietnam	Reservoir water	2021	[110]
Vietnam	Fish and other seafood	2017	[111]
Vietnam	Reservoir water	2017	[112]
Vietnam	Reservoir water	2018	[113]
Vietnam	Reservoir water	2020	[114]
Vietnam	Fish and other seafood	2018	[115]

References

1. Gevorgyan, G.; Rinke, K.; Schultze, M.; Mamyan, A.; Kuzmin, A.; Belykh, O.; Sorokovikova, E.; Hayrapetyan, A.; Hovsepyan, A.; Khachikyan, T.; et al. First report about toxic cyanobacterial bloom occurrence in Lake Sevan, Armenia. *International Review of Hydrobiology* **2020**, *105*, 131–142, doi:10.1002/iroh.202002060.
2. Minasyan, A.; Christophoridis, C.; Wilson, A.E.; Zervou, S.K.; Kaloudis, T.; Hiskia, A. Diversity of cyanobacteria and the presence of cyanotoxins in the epilimnion of Lake Yerevan (Armenia). *Toxicon* **2018**, *150*, 28–38, doi:10.1016/j.toxicon.2018.04.021.
3. Affan, A.; Khomayis, H.S.; Al-Harbi, S.M.; Haqueand, M.; Khan, S. Effect of environmental factors on cyanobacteria i abundance and cyanotoxins production in natural and drinking water, Bangladesh. *Pakistan Journal of Biological Sciences* **2015**, *18*, 50–58.
4. Sagir Ahmed, M. Isolation and Characterization of Microcystins (Heptapeptides Hepatotoxins) from Microcystis aeruginosa Bloom in a Homestead Pond, Dhaka, Bangladesh. *Research Journal of Environmental Sciences* **2009**, *3*, 245–250, doi:10.3923/rjes.2009.245.250.
5. Jahan, R.; Khan, S.; Haque, M.M.; Choi, J.K. Study of harmful algal blooms in a eutrophic pond, Bangladesh. *Environmental Monitoring and Assessment* **2010**, *170*, 7–21, doi:10.1007/s10661-009-1210-4.
6. Bi, X.; Dai, W.; Wang, X.; Dong, S.; Zhang, S.; Zhang, D.; Wu, M. Microcystins distribution, bioaccumulation, and Microcystis genotype succession in a fish culture pond. *Science of the Total Environment* **2019**, *688*, 380–388, doi:10.1016/j.scitotenv.2019.06.156.
7. Cao, Q.; Steinman, A.D.; Wan, X.; Xie, L. Bioaccumulation of microcystin congeners in soil-plant system and human health risk assessment: A field study from Lake Taihu region of China. *Environmental Pollution* **2018**, *240*, 44–50, doi:10.1016/j.envpol.2018.04.067.
8. Chen, J.; Xie, P. Accumulation of hepatotoxic microcystes in freshwater mussels, aquatic insect larvae and oligochaetes in a large, shallow eutrophic lake (Lake Chaohu) of subtropical China. *Fresenius Environmental Bulletin* **2008**, *17*, 849–854.
9. Chen, J.; Xie, P. Tissue distributions and seasonal dynamics of the hepatotoxic microcystins-LR and -RR in two freshwater shrimps, Palaemon modestus and Macrobrachium nipponensis, from a large shallow, eutrophic lake of the subtropical China. *Toxicon* **2005**, *45*, 615–625, doi:10.1016/j.toxicon.2005.01.003.
10. Chen, J.; Xie, P. Seasonal dynamics of the hepatotoxic microcystins in various organs of four freshwater bivalves from the large eutrophic Lake Taihu of subtropical China and the risk to human consumption. *Environmental Toxicology* **2005**, *20*, 572–584, doi:10.1002/tox.20146.
11. Chen, J.; Xie, P.; Zhang, D.; Ke, Z.; Yang, H. In situ studies on the bioaccumulation of microcystins in the phytoplanktivorous silver carp (Hypophthalmichthys molitrix) stocked in Lake Taihu with dense toxic Microcystis blooms. *Aquaculture* **2006**, *261*, 1026–1038, doi:10.1016/j.aquaculture.2006.08.028.

12. Chen, J.; Xie, P.; Zhang, D.; Lei, H. In situ studies on the distribution patterns and dynamics of microcystins in a biomanipulation fish - bighead carp (Aristichthys nobilis). *Environmental Pollution* **2007**, *147*, 150–157, doi:10.1016/j.envpol.2006.08.015.
13. Chen, J.; Zhang, D.; Xie, P.; Wang, Q.; Ma, Z. Simultaneous determination of microcystin contaminations in various vertebrates (fish, turtle, duck and water bird) from a large eutrophic Chinese lake, Lake Taihu, with toxic Microcystis blooms. *Science of the Total Environment* **2009**, *407*, 3317–3322, doi:10.1016/j.scitotenv.2009.02.005.
14. Dai, R.; Liu, H.; Qu, J.; Ru, J.; Hou, Y. Cyanobacteria and their toxins in Guanting Reservoir of Beijing, China. *Journal of Hazardous Materials* **2008**, *153*, 470–477, doi:10.1016/j.jhazmat.2007.08.078.
15. Hu, L.; Shan, K.; Lin, L.; Shen, W.; Huang, L.; Gan, N.; Song, L. Multi-year assessment of toxic genotypes and microcystin concentration in northern lake taihu, China. *Toxins* **2016**, *8*, doi:10.3390/toxins8010023.
16. He, Q.; Kang, L.; Sun, X.; Jia, R.; Zhang, Y.; Ma, J.; Li, H.; Ai, H. Spatiotemporal distribution and potential risk assessment of microcystins in the Yulin River, a tributary of the Three Gorges Reservoir, China. *Journal of Hazardous Materials* **2018**, *347*, 184–195, doi:10.1016/j.jhazmat.2018.01.001.
17. Jia, J.; Luo, W.; Lu, Y.; Giesy, J.P. Bioaccumulation of microcystins (MCs) in four fish species from Lake Taihu, China: Assessment of risks to humans. *Science of the Total Environment* **2014**, *487*, 224–232, doi:10.1016/j.scitotenv.2014.04.037.
18. Jiang, Y.; Xie, P.; Nie, Y. Concentration and bioaccumulation of cyanobacterial bioactive and odorous metabolites occurred in a large, shallow chinese lake. *Bulletin of Environmental Contamination and Toxicology* **2014**, *93*, 643–648, doi:10.1007/s00128-014-1350-2.
19. Jiang, Y.; Yang, Y.; Wu, Y.; Tao, J.; Cheng, B. Microcystin Bioaccumulation in Freshwater Fish at Different Trophic Levels from the Eutrophic Lake Chaohu, China. *Bulletin of Environmental Contamination and Toxicology* **2017**, *99*, 69–74, doi:10.1007/s00128-017-2047-0.
20. Jing, M.; Lin, D.; Lin, J.; Li, Q.; Yan, H.; Feng, X. Mercury, microcystins and Omega-3 polyunsaturated fatty acids in farmed fish in eutrophic reservoir: Risk and benefit assessment. *Environmental Pollution* **2021**, *270*, 116047, doi:10.1016/j.envpol.2020.116047.
21. Lei, L.; Peng, L.; Huang, X.; Han, B.P. Occurrence and dominance of Cylindrospermopsis raciborskii and dissolved cylindrospermopsin in urban reservoirs used for drinking water supply, South China. *Environmental Monitoring and Assessment* **2014**, *186*, 3079–3090, doi:10.1007/s10661-013-3602-8.
22. Li, S.; Xie, P.; Xu, J.; Zhang, X.; Qin, J.; Zheng, L.; Liang, G. Factors shaping the pattern of seasonal variations of microcystins in Lake Xingyun, a subtropical plateau lake in China. *Bulletin of Environmental Contamination and Toxicology* **2007**, *78*, 226–230, doi:10.1007/s00128-007-9116-8.
23. Li, D.; Erickson, R.A.; Tang, S.; Zhang, Y.; Niu, Z.; Liu, H.; Yu, H. Structure and spatial patterns of macrobenthic community in Tai Lake, a large shallow lake, China. *Ecological Indicators* **2016**, *61*, 179–187, doi:10.1016/j.ecolind.2015.08.043.
24. Li, D. ming; Zheng, H. yan; Pan, J. lin; Zhang, T. qing; Tang, S. kai; Lu, J. ming; Zhong, L. qiang; Liu, Y. shan; Liu, X. wei Seasonal dynamics of photosynthetic activity, Microcystis genotypes and microcystin production in Lake Taihu, China. *Journal of Great Lakes Research* **2017**, *43*, 710–716, doi:10.1016/j.jglr.2017.04.005.
25. Ni, W.; Zhang, J.; Luo, Y. Microcystin accumulation in bighead carp (Aristichthys nobilis) during a Microcystis-dominated bloom and risk assessment of the dietary intake in a fish pond in China. *Environmental Science and Pollution Research* **2017**, *24*, 8894–8902, doi:10.1007/s11356-015-4974-9.
26. Peng, L.; Liu, Y.; Chen, W.; Liu, L.; Kent, M.; Song, L. Health risks associated with consumption of microcystin-contaminated fish and shellfish in three Chinese lakes: Significance for freshwater aquacultures. *Ecotoxicology and Environmental Safety* **2010**, *73*, 1804–1811, doi:10.1016/j.ecoenv.2010.07.043.
27. Peng, L.; Tang, Q.; Gu, J.; Lei, L.; Chen, W.; Song, L. Seasonal variation of microcystins and their accumulation in fish in two large shallow lakes of China. *Ecotoxicology* **2020**, *29*, 790–800, doi:10.1007/s10646-020-02231-2.
28. Qiu, T.; Xie, P.; Li, L.; Guo, L.; Zhang, D.; Zhou, Q. Nephrotoxic effects from chronic toxic cyanobacterial blooms in fishes with different trophic levels in a large Chinese lake. *Environmental Toxicology and Pharmacology* **2012**, *33*, 252–261, doi:10.1016/j.etap.2011.12.004.
29. Sakai, H.; Hao, A.; Iseri, Y.; Wang, S.; Kuba, T.; Zhang, Z.; Katayama, H. Occurrence and distribution of microcystins in Lake Taihu, China. *The Scientific World Journal* **2013**, *2013*, doi:10.1155/2013/838176.
30. Shang, L.; Feng, M.; Liu, F.; Xu, X.; Ke, F.; Chen, X.; Li, W. The establishment of preliminary safety threshold values for cyanobacteria based on periodic variations in different microcystin congeners in Lake Chaohu, China. *Environmental Sciences: Processes and Impacts* **2015**, *17*, 728–739,

doi:10.1039/c5em00002e.

31. Shen, P.P.; Shi, Q.; Hua, Z.C.; Kong, F.X.; Wang, Z.G.; Zhuang, S.X.; Chen, D.C. Analysis of microcystins in cyanobacteria blooms and surface water samples from Meiliang Bay, Taihu Lake, China. *Environment International* **2003**, *29*, 641–647, doi:10.1016/S0160-4120(03)00047-3.
32. Su, X.; Xue, Q.; Steinman, A.D.; Zhao, Y.; Xie, L. Spatiotemporal dynamics of microcystin variants and relationships with environmental parameters in lake Taihu, China. *Toxins* **2015**, *7*, 3224–3244, doi:10.3390/toxins7083224.
33. Su, X.; Steinman, A.D.; Xue, Q.; Zhao, Y.; Xie, L. Evaluating the contamination of microcystins in Lake Taihu, China: The application of equivalent total MC-LR concentration. *Ecological Indicators* **2018**, *89*, 445–454, doi:10.1016/j.ecolind.2017.11.042.
34. Tian, D.; Zheng, W.; Wei, X.; Sun, X.; Liu, L.; Chen, X.; Zhang, H.; Zhou, Y.; Chen, H.; Zhang, H.; et al. Dissolved microcystins in surface and ground waters in regions with high cancer incidence in the Huai River Basin of China. *Chemosphere* **2013**, *91*, 1064–1071, doi:10.1016/j.chemosphere.2013.01.051.
35. Wan, X.; Steinman, A.D.; Gu, Y.; Zhu, G.; Shu, X.; Xue, Q.; Zou, W.; Xie, L. Occurrence and risk assessment of microcystin and its relationship with environmental factors in lakes of the eastern plain ecoregion, China. *Environmental Science and Pollution Research* **2020**, *27*, 45095–45107, doi:10.1007/s11356-020-10384-0.
36. Wang, Q.; Niu, Y.; Xie, P.; Chen, J.; Ma, Z.; Tao, M.; Qi, M.; Wu, L.; Guo, L. Factors affecting temporal and spatial variations of microcystins in Gonghu Bay of Lake Taihu, with potential risk of microcystin contamination to human health. *TheScientificWorldJournal* **2010**, *10*, 1795–1809, doi:10.1100/tsw.2010.172.
37. Wu, S.K.; Xie, P.; Liang, G.D.; Wang, S.B.; Liang, X.M. Relationships between microcystins and environmental parameters in 30 subtropical shallow lakes along the Yangtze River, China. *Freshwater Biology* **2006**, *51*, 2309–2319, doi:10.1111/j.1365-2427.2006.01652.x.
38. Xiao, C.C.; Chen, M.J.; Mei, F.B.; Fang, X.; Huang, T.R.; Li, J.L.; Deng, W.; Li, Y.D. Influencing factors and health risk assessment of microcystins in the Yongjiang river (China) by Monte Carlo simulation. *PeerJ* **2018**, *2018*, 1–21, doi:10.7717/peerj.5955.
39. Xie, L.; Xie, P.; Guo, L.; Li, L.; Miyabara, Y.; Park, H.D. Organ distribution and bioaccumulation of microcystins in freshwater fish at different trophic levels from the eutrophic Lake Chaohu, China. *Environmental Toxicology* **2005**, *20*, 293–300, doi:10.1002/tox.20120.
40. Xu, C.; Chen, J.A.; Huang, Y.J.; Qiu, Z.Q.; Luo, J.H.; Zeng, H.; Zhao, Q.; Cao, J.; Shu, W.Q. Identification of microcystins contamination in surface water samples from the Three Gorges Reservoir, China. *Environmental Monitoring and Assessment* **2011**, *180*, 77–86, doi:10.1007/s10661-010-1773-0.
41. Xue, Q.; Steinman, A.D.; Su, X.; Zhao, Y.; Xie, L. Temporal dynamics of microcystins in Limnodrilus hoffmeisteri, a dominant oligochaete of hypereutrophic Lake Taihu, China. *Environmental Pollution* **2016**, *213*, 585–593, doi:10.1016/j.envpol.2016.03.043.
42. Xue, Q.; Rediske, R.R.; Gong, Z.; Su, X.; Xu, H.; Cai, Y.; Zhao, Y.; Xie, L. Spatio-temporal variation of microcystins and its relationship to biotic and abiotic factors in Hongze Lake, China. *Journal of Great Lakes Research* **2018**, *44*, 253–262, doi:10.1016/j.jglr.2017.12.004.
43. Xue, Q.; Steinman, A.D.; Xie, L.; Yao, L.; Su, X.; Cao, Q.; Zhao, Y.; Cai, Y. Seasonal variation and potential risk assessment of microcystins in the sediments of Lake Taihu, China. *Environmental Pollution* **2020**, *259*, 113884, doi:10.1016/j.envpol.2019.113884.
44. Yang, H.; Xie, P.; Xu, J.; Zheng, L.; Deng, D.; Zhou, Q.; Wu, S. Seasonal variation of microcystin concentration in lake Chaohu, a shallow subtropical lake in the People's Republic of China. *Bulletin of Environmental Contamination and Toxicology* **2006**, *77*, 367–374, doi:10.1007/s00128-006-1075-y.
45. Yang, Z.; Kong, F.; Zhang, M. Groundwater contamination by microcystin from toxic cyanobacteria blooms in Lake Chaohu, China. *Environmental Monitoring and Assessment* **2016**, *188*, doi:10.1007/s10661-016-5289-0.
46. Yen, H.K.; Lin, T.F.; Liao, P.C. Simultaneous detection of nine cyanotoxins in drinking water using dual solid-phase extraction and liquid chromatography-mass spectrometry. *Toxicon* **2011**, *58*, 209–218, doi:10.1016/j.toxicon.2011.06.003.
47. Yu, L.; Kong, F.; Zhang, M.; Yang, Z.; Shi, X.; Du, M. The dynamics of microcystis genotypes and microcystin production and associations with environmental factors during blooms in lake chaohu, china. *Toxins* **2014**, *6*, 3238–3257, doi:10.3390/toxins6123238.
48. Zhang, H. jun; Zhang, J. ying; Hong, Y.; Chen, Y. xu Evaluation of organ distribution of microcystins in the freshwater phytoplanktivorous fish Hypophthalmichthys molitrix. *Journal of Zhejiang University. Science. B.* **2007**, *8*, 116–120.

49. Zhang, H.; Zhang, J.; Zhu, Y. Identification of Microcystins in waters used for daily life by people who live on Tai Lake during a serious cyanobacteria dominated bloom with risk analysis to human health. *Environmental Toxicology* **2008**, *24*, 82–86, doi:10.1002/tox.20381.
50. Zhang, D.; Xie, P.; Liu, Y.; Chen, J.; Wen, Z. Spatial and temporal variations of microcystins in hepatopancreas of a freshwater snail from Lake Taihu. *Ecotoxicology and Environmental Safety* **2009**, *72*, 466–472, doi:10.1016/j.ecoenv.2008.05.014.
51. Zhang, D.; Xie, P.; Liu, Y.; Qiu, T. Transfer, distribution and bioaccumulation of microcystins in the aquatic food web in Lake Taihu, China, with potential risks to human health. *Science of the Total Environment* **2009**, *407*, 2191–2199.
52. Zhang, H.; Jia, X.; Hu, C. Environmental factors influencing seasonal variation of microcystin-RR concentrations in Baoyang River, a shallow city river in Changxing of China. *2010 4th International Conference on Bioinformatics and Biomedical Engineering, iCBBE 2010* **2010**, 8–10, doi:10.1109/ICBBE.2010.5518212.
53. Zhang, J.; Wang, Z.; Song, Z.; Xie, Z.; Li, L.; Song, L. Bioaccumulation of microcystins in two freshwater gastropods from a cyanobacteria-bloom plateau lake, Lake Dianchi. *Environmental Pollution* **2012**, *164*, 227–234.
54. Zhang, D.; Deng, X.; Xie, P.; Chen, J.; Guo, L. Risk assessment of microcystins in silver carp (Hypophthalmichthys molitrix) from eight eutrophic lakes in China. *Food Chemistry* **2013**, *140*, 17–21.
55. Zhang, D.; Liao, Q.; Zhang, L.; Wang, D.; Luo, L.; Chen, Y.; Zhong, J.; Liu, J. Occurrence and spatial distributions of microcystins in Poyang Lake, the largest freshwater lake in China. *Ecotoxicology* **2015**, *24*, 19–28, doi:10.1007/s10646-014-1349-9.
56. Zhang, L.; Liu, J.T.; Zhang, D.; Luo, L.; Liao, Q.; Yuan, L.; Wu, N. Seasonal and Spatial Variations of Microcystins and Their Relationships with Physiochemical and Biological Factors in Poyang Lake. *Huanjing Kexue/Environmental Science* **2018**, *39*, 450–459, doi:10.13227/j.hjkx.201708227.
57. Zhao, M.; Xie, S.; Zhu, X.; Yang, Y.; Gan, N.; Song, L. Effect of dietary cyanobacteria on growth and accumulation of microcystins in Nile tilapia (Oreochromis niloticus). *Aquaculture* **2006**, *261*, 960–966, doi:10.1016/j.aquaculture.2006.08.019.
58. Zhu, R.; Wang, H.; Shen, H.; Deng, X.; Chen, J. The dynamics and release characteristics of microcystins in the plateau Lake Erhai, Southwest China. *Environmental Science and Pollution Research* **2021**, *28*, 23473–23481, doi:10.1007/s11356-020-12312-8.
59. Chaturvedi, P.; Kumar Agrawal, M.; Nath Bagchi, S. Microcystin-producing and non-producing cyanobacterial blooms collected from the Central India harbor potentially pathogenic Vibrio cholerae. *Ecotoxicology and Environmental Safety* **2015**, *115*, 67–74, doi:10.1016/j.ecoenv.2015.02.001.
60. Dixit, R.B.; Patel, A.K.; Toppo, K.; Nayaka, S. Emergence of toxic cyanobacterial species in the Ganga River, India, due to excessive nutrient loading. *Ecological Indicators* **2017**, *72*, 420–427, doi:10.1016/j.ecolind.2016.08.038.
61. Agrawal, M.K.; Ghosh, S.K.; Bagchi, D.; Weckesser, J.; Erhard, M.; Bagchi, S.N. Occurrence of microcystin-containing toxic water blooms in Central India. *Journal of Microbiology and Biotechnology* 2006, *16*, 212–218.
62. Maske, S.S.; Sangolkar, L.N.; Chakrabarti, T. Temporal variation in density and diversity of cyanobacteria and cyanotoxins in lakes at Nagpur (Maharashtra State), India. *Environmental Monitoring and Assessment* **2010**, *169*, 299–308, doi:10.1007/s10661-009-1171-7.
63. Sangolkar, L.N.; Maske, S.S.; Muthal, P.L.; Kashyap, S.M.; Chakrabarti, T. Isolation and characterization of microcystin producing Microcystis from a Central Indian water bloom. *Harmful Algae* **2009**, *8*, 674–684, doi:10.1016/j.hal.2008.12.003.
64. Singh, S.; Asthana, R.K. Assessment of microcystin concentration in carp and catfish: A case study from Lakshmikund Pond, Varanasi, India. *Bulletin of Environmental Contamination and Toxicology* **2014**, *92*, 687–692.
65. Singh, S.; Rai, P.K.; Chau, R.; Ravi, A.K.; Neilan, B.A.; Asthana, R.K. Temporal variations in microcystin-producing cells and microcystin concentrations in two fresh water ponds. *Water Research* **2015**, *69*, 131–142, doi:10.1016/j.watres.2014.11.015.
66. Rezaitabar, S.; Esmaili Sari, A.; Bahramifar, N.; Ramezanpour, Z. Transfer, tissue distribution and bioaccumulation of microcystin LR in the phytoplanktivorous and carnivorous fish in Anzali wetland, with potential health risks to humans. *Science of the Total Environment* **2017**, *575*, 1130–1138, doi:10.1016/j.scitotenv.2016.09.199.
67. Baldia, S.F.; Conaco, M.C.G.; Nishijima, T.; Imanishi, S.; Harada, K.I. Microcystin production during

algal bloom occurrence in Laguna de Bay, the Philippines. *Fisheries Science* **2003**, *69*, 110–116, doi:10.1046/j.1444-2906.2003.00594.x.
68. Babanazarova, O. V.; Kurmayer, R.; Sidelev, S.I.; Aleksandrina, E.M.; Sakharova, E.G. Phytoplankton structure and microcystine concentration in the highly eutrophic Nero Lake. *Water Resources* **2011**, *38*, 229–236, doi:10.1134/S0097807811020023.
69. Belykh, O.I.; Sorokovikova, E.G.; Fedorova, G.A.; Kaluzhnaya, O. V.; Korneva, E.S.; Sakirko, M. V.; Sherbakova, T.A. Presence and genetic diversity of microcystin-producing cyanobacteria (Anabaena and Microcystis) in Lake Kotokel (Russia, Lake Baikal Region). *Hydrobiologia* **2011**, *671*, 241–252, doi:10.1007/s10750-011-0724-2.
70. Belykh, O.I.; Gladkikh, A.S.; Sorokovikova, E.G.; Tikhonova, I. V.; Potapov, S.A.; Butina, T. V. Saxitoxin-Producing cyanobacteria in Lake Baikal. *Contemporary Problems of Ecology* **2015**, *8*, 186–192, doi:10.1134/S199542551502002X.
71. Belykh, O.I.; Glagkikh, A.S.; Tikhonova, I. V.; Kuz'min, A. V.; Mogil'nikova, T.A.; Fedorova, G.A.; Sorokovikova, E.G. Identification of cyanobacterial producers of shellfish paralytic toxins in lake Baikal and reservoirs of the Angara River. *Microbiology (Russian Federation)* **2015**, *84*, 98–99, doi:10.1134/S0026261715010038.
72. Belykh, O.I.; Tikhonova, I. V.; Kuzmin, A. V.; Sorokovikova, E.G.; Fedorova, G.A.; Khanaev, I. V.; Sherbakova, T.A.; Timoshkin, O.A. First detection of benthic cyanobacteria in Lake Baikal producing paralytic shellfish toxins. *Toxicon* **2016**, *121*, 36–40, doi:10.1016/j.toxicon.2016.08.015.
73. Chernova, E.; Russkikh, I.; Voyakina, E.; Zhakovskaya, Z. Occurrence of microcystins and anatoxin-a in eutrophic lakes of Saint Petersburg, Northwestern Russia. *Oceanological and Hydrobiological Studies* **2016**, *45*, 466–484, doi:10.1515/ohs-2016-0040.
74. Chernova, E.; Sidelev, S.; Russkikh, I.; Voyakina, E.; Babanazarova, O.; Romanov, R.; Kotovshchikov, A.; Mazur-Marzec, H. Dolichospermum and Aphanizomenon as neurotoxins producers in some Russian freshwaters. *Toxicon* **2017**, *130*, 47–55, doi:10.1016/j.toxicon.2017.02.016.
75. Chernova, E.; Sidelev, S.; Russkikh, I.; Korneva, L.; Solovyova, V.; Mineeva, N.; Stepanova, I.; Zhakovskaya, Z. Spatial distribution of cyanotoxins and ratios of microcystin to biomass indicators in the reservoirs of the Volga, Kama and Don Rivers, the European part of Russia. *Limnologica* **2020**, *84*, 125819, doi:10.1016/j.limno.2020.125819.
76. Grachev, M.; Zubkov, I.; Tikhonova, I.; Ivacheva, M.; Kuzmin, A.; Sukhanova, E.; Sorokovikova, E.; Fedorova, G.; Galkin, A.; Suslova, M.; et al. Extensive contamination of water with saxitoxin near the dam of the irkutsk hydropower station reservoir (East siberia, russia). *Toxins* **2018**, *10*, 1. – 12, doi:10.3390/toxins10100402.
77. Nikitin, O. V.; Stepanova, N.Y.; Latypova, V.Z. Human health risk assessment related to blue-green algae mass development in the Kuibyshev Reservoir. *Water Science and Technology: Water Supply* **2015**, *15*, 693–700, doi:10.2166/ws.2015.022.
78. Sidelev, S.I.; Babanazarova, O. V. Detection of Cyanobacterial Toxins in Water Supply Sources and Tap Water in Some Russian Cities: Searching Producers and Testing Removal Methods. *Water Resources* **2020**, *47*, 304–314, doi:10.1134/S0097807820020189.
79. Sidelev, S.I.; Fomichev, A.A.; Babanazarova, O. V.; Zubishina, A.A. Detection of microcystin-producing cyanobacteria in the upper Volga reservoirs. *Microbiology (Russian Federation)* **2013**, *82*, 387–388, doi:10.1134/S0026261713020148.
80. Sidelev, S.I.; Golokolenova, T.B.; Chernova, E.N.; Russkikh, Y. V. Analysis of phytoplankton in Tsimlyansk Reservoir (Russia) for the presence of cyanobacterial hepato- and neurotoxins. *Microbiology (Russian Federation)* **2015**, *84*, 828–837, doi:10.1134/S0026261715060120.
81. Jayatissa, L.P.; Silva, E.I.L.; McElhiney, J.; Lawton, L.A. Occurrence of toxigenic cyanobacterial blooms in freshwaters of Sri Lanka. *Systematic and Applied Microbiology* **2006**, *29*, 156–164, doi:10.1016/j.syapm.2005.07.007.
82. Li, R.; Carmichael, W.W.; Brittain, S.; Eaglesham, G.K.; Shaw, G.R.; Mahakhant, A.; Noparatnaraporn, N.; Yongmanitchai, W.; Kaya, K.; Watanabe, M.M. Isolation and identification of the cyanotoxin cylindrospermopsin and deoxy-cylindrospermopsin from a Thailand strain of Cylindrospermopsis raciborskii (Cyanobacteria). *Toxicon* **2001**, *39*, 973–980, doi:10.1016/S0041-0101(00)00236-1.
83. Ruangrit, K.; Whangchai, N.; Pekkoh, J.; Ruangyuttikarn, W.; Peerapornpisal, Y. First report on microcystins contamination in giant freshwater prawn (Macrobrachium rosenbergii) and Nile tilapia (Tilapia nilotica) cultured in earthen ponds. *International Journal of Agriculture and Biology* **2011**, *13*, 1025–1028.
84. Ruangrit, K.; Peerapornpisal, Y.; Pekkoh, J.; Whangchai, N. Microcystin Accumulation in Nile Tilapia,

Oreochromis niloticus and Giant Freshwater Prawns, Macrobrachium rosenbergii in Green Water System Cultivation. *International Journal of Geosciences* **2013**, *04*, 60–63, doi:10.4236/ijg.2013.45b010.
85. Ruangsomboon, S.; Yongmanitchai, W.; Taveekijakarn, P.; Ganmanee, M. Cyanobacterial composition and microcystin accumulation in catfish pond. *Chiang Mai Journal of Science* **2014**, *41*, 27–38.
86. Sengpracha, W.; Suvannachai, N.; Phutdhawong, W. Microcystin LR Content in Microcystis aeruginosa Kütz Collected from Sri Sakhett, Thailand. *Chiang Mai Journal of Science* **2006**, *33*, 231–236.
87. Somdee, T.; Kaewsan, T.; Somdee, A. Monitoring toxic cyanobacteria and cyanotoxins (microcystins and cylindrospermopsins) in four recreational reservoirs (Khon Kaen, Thailand). *Environmental Monitoring and Assessment* **2013**, *185*, 9521–9529, doi:10.1007/s10661-013-3270-8.
88. Wang, X.; Parkpian, P.; Fujimoto, N.; Ruchirawat, K.M.; DeLaune, R.D.; Jugsujinda, A. Environmental conditions associating microcystins production to Microcystis aeruginosa in a reservoir of Thailand. *Journal of Environmental Science and Health - Part A Toxic/Hazardous Substances and Environmental Engineering* **2002**, *37*, 1181–1207, doi:10.1081/ESE-120005980.
89. Whangchai, N.; Wanno, S.; Gutierrez, R.; Kannika, K.; Promna, R.; Iwami, N.; Itayama, T. Accumulation of microcystins in water and economic fish in Phayao Lake, and fish ponds along the Ing River tributary in Chiang Rai, Thailand. *Agricultural Sciences* **2013**, *04*, 52–56, doi:10.4236/as.2013.45b010.
90. Akcaalan, R.; Mazur-Marzec, H.; Zalewska, A.; Albay, M. Phenotypic and toxicological characterization of toxic Nodularia spumigena from a freshwater lake in Turkey. *Harmful Algae* **2009**, *8*, 273–278, doi:10.1016/j.hal.2008.06.007.
91. Akçaalan, R.; Köker, L.; Gürevin, C.; Albay, M. Planktothrix rubescens: A perennial presence and toxicity in Lake Sapanca. *Turkish Journal of Botany* **2014**, *38*, 782–789, doi:10.3906/bot-1401-26.
92. Akcaalan, R.; Köker, L.; Oğuz, A.; Spoof, L.; Meriluoto, J.; Albay, M. First report of cylindrospermopsin production by two cyanobacteria (Dolichospermum mendotae and Chrysosporum ovalisporum) in Lake Iznik, Turkey. *Toxins* **2014**, *6*, 3173–3186.
93. Akçaalan, R.; Albay, M.; Koker, L.; Baudart, J.; Guillebault, D.; Fischer, S.; Weigel, W.; Medlin, L.K. Seasonal dynamics of freshwater pathogens as measured by microarray at Lake Sapanca, a drinking water source in the north-eastern part of Turkey. *Environmental Monitoring and Assessment* **2018**, *190*, doi:10.1007/s10661-017-6314-7.
94. Albay, M.; Akcaalan, R.; Aykulu, G.; Tufekci, H.; Beattie, K.A.; Codd, G.A. Occurrence of toxic cyanobacteria before and after copper sulphate treatment in a water reservoir, Istanbul, Turkey. *Algological Studies* **2009**, *109*, 67–78, doi:10.1127/1864-1318/2003/0109-0067.
95. Albay, M.; Akcaalan, R.; Tufekci, H.; Metcalf, J.S.; Beattie, K.A.; Codd, G.A. Depth profiles of cyanobacterial hepatotoxins (microcystins) in three Turkish freshwater lakes. *Hydrobiologia* **2003**, *505*, 89–95, doi:10.1023/B:HYDR.0000007297.29998.5f.
96. Albay, M.; Matthiensen, A.; Codd, G.A. Occurrence of toxic blue-green algae in the Kucukcekmece Lagoon (Istanbul, Turkey). *Environmental Toxicology* **2005**, *20*, 277–284, doi:10.1002/tox.20118.
97. Gurbuz, F.; Metcalf, J.S.; Karahan, A.G.; Codd, G.A. Analysis of dissolved microcystins in surface water samples from Kovada Lake, Turkey. *Science of the Total Environment* **2009**, *407*, 4038–4046, doi:10.1016/j.scitotenv.2009.02.039.
98. Gurbuz, F.; Uzunmehmetoğlu, O.Y.; Diler, Ö.; Metcalf, J.S.; Codd, G.A. Occurrence of microcystins in water, bloom, sediment and fish from a public water supply. *Science of the Total Environment* **2016**, *562*, 860–868, doi:10.1016/j.scitotenv.2016.04.027.
99. Sahindokuyucu Kocasari, F.; Gulle, I.; Kocasari, S.; Pekkaya, S.; Mor, F. The occurrence and levels of cyanotoxin nodularin from nodularia spumigena in the alkaline and salty Lake Burdur, Turkey. *Journal of Limnology* **2015**, *74*, 530–536, doi:10.4081/jlimnol.2015.1097.
100. Köker, L.; Akçaalan, R.; Dittmann, E.; Albay, M. Depth profiles of protein-bound microcystin in Küçükçekmece Lagoon. *Toxicon* **2021**, *198*, 156–163, doi:10.1016/j.toxicon.2021.05.005.
101. Köker, L.; Akçaalan, R.; Albay, M.; Neilan, B.A. Molecular detection of hepatotoxic cyanobacteria in inland water bodies of the Marmara region, Turkey. *Advances in Oceanography and Limnology* **2017**, *8*, 52–60, doi:10.4081/aiol.2017.6394.
102. Koker, L.; Akcaalan, R.; Oguz, A.; Gaygusuz, O.; Kose, C.A.; Gucver, S.; Karaaslan, Y.; Albay, M.; Kinaci, C. Distribution of toxic cyanobacteria and cyanotoxins in Turkish waterbodies. *Journal of Environmental Protection and Ecology* **2017**, *18*, 425–432.
103. Rodriguez, I.; Fraga, M.; Alfonso, A.; Guillebault, D.; Medlin, L.; Baudart, J.; Jacob, P.; Helmi, K.; Meyer, T.; Breitenbach, U.; et al. Monitoring of freshwater toxins in European environmental waters by using novel multi-detection methods. *Environmental Toxicology and Chemistry* **2017**, *36*, 645–654,

doi:10.1002/etc.3577.
104. Yilmaz, M.; Foss, A.J.; Selwood, A.I.; Özen, M.; Boundy, M. Paralytic shellfish toxin producing Aphanizomenon gracile strains isolated from Lake Iznik, Turkey. *Toxicon* **2018**, *148*, 132–142, doi:10.1016/j.toxicon.2018.04.028.
105. Yilmaz, M.; Foss, A.J.; Miles, C.O.; Özen, M.; Demir, N.; Balcı, M.; Beach, D.G. Comprehensive multi-technique approach reveals the high diversity of microcystins in field collections and an associated isolate of Microcystis aeruginosa from a Turkish lake. *Toxicon* **2019**, *167*, 87–100, doi:10.1016/j.toxicon.2019.06.006.
106. Dao, T.S.; Cronberg, G.; Nimptsch, J.; Do-Hong, L.C.; Wiegand, C. Toxic cyanobacteria from Tri An Reservoir, Vietnam. *Nova Hedwigia* **2010**, *90*, 433–448, doi:10.1127/0029-5035/2010/0090-0433.
107. Duong, T.T.; Le, T.P.Q.; Dao, T.S.; Pflugmacher, S.; Rochelle-Newall, E.; Hoang, T.K.; Vu, T.N.; Ho, C.T.; Dang, D.K. Seasonal variation of cyanobacteria and microcystins in the Nui Coc Reservoir, Northern Vietnam. *Journal of Applied Phycology* **2013**, *25*, 1065–1075, doi:10.1007/s10811-012-9919-9.
108. Duong, T.T.; Jähnichen, S.; Le, T.P.Q.; Ho, C.T.; Hoang, T.K.; Nguyen, T.K.; Vu, T.N.; Dang, D.K. The occurrence of cyanobacteria and microcystins in the Hoan Kiem Lake and the Nui Coc reservoir (North Vietnam). *Environmental Earth Sciences* **2014**, *71*, 2419–2427, doi:10.1007/s12665-013-2642-2.
109. Hummert, C.; Dahlmann, J.; Reinhardt, K.; Dang, H.P.H.; Dang, D.K.; Luckas, B. Liquid chromatography - Mass spectrometry identification of microcystins in Microcystis aeruginosa strain from lake Thanh Cong, Hhanoi, Vietnam. *Chromatographia* **2001**, *54*, 569–575, doi:10.1007/BF02492180.
110. Nguyen, T.A.D.; Nguyen, L.T.; Enright, A.; Pham, L.T.; Tran, H.Y.T.; Tran, T.T.; Nguyen, V.H.T.; Tran, D.N. Health risk assessment related to cyanotoxins exposure of a community living near Tri An Reservoir, Vietnam. *Environmental Science and Pollution Research* **2021**, doi:10.1007/s11356-021-14545-7.
111. Pham, T.L.; Shimizu, K.; Dao, T.S.; Motoo, U. First report on free and covalently bound microcystins in fish and bivalves from Vietnam: Assessment of risks to humans. *Environmental Toxicology and Chemistry* **2017**, *36*, 2953–2957, doi:10.1002/etc.3858.
112. Pham, T.L.; Dao, T.S.; Tran, N.D.; Nimptsch, J.; Wiegand, C.; Motoo, U. Influence of environmental factors on cyanobacterial biomass and microcystin concentration in the Dau Tieng Reservoir, a tropical eutrophic water body in Vietnam. *Annales de Limnologie* **2017**, *53*, 89–100, doi:10.1051/limn/2016038.
113. Luu, P.T. Diversity of microcystins and non-microcystin-producing *Microcystics* population in the Dau Tieng reservoir, Vietnam. *Academia Journal of Biology* **2018**, *40*, 57–66, doi:10.15625/2615-9023/v40n4.10265.
114. Pham, T.L.; Tran, T.H.Y.; Shimizu, K.; Li, Q.; Utsumi, M. Toxic cyanobacteria and microcystin dynamics in a tropical reservoir: assessing the influence of environmental variables. *Environmental Science and Pollution Research* **2020**, doi:10.1007/s11356-020-10826-9.
115. Trung, B.; Dao, T.S.; Faassen, E.; Lürling, M. Cyanobacterial blooms and microcystins in Southern Vietnam. *Toxins* **2018**, *10*, 1–20, doi:10.3390/toxins10110471.
