# Supplementary material for: Cyanotoxins and Food Contamination in Developing Countries: Review of Their Types, Toxicity, Analysis, Occurrence and Mitigation Strategies
Source: Toxins (Basel). 2021 Nov 6;13(11):786. doi: 10.3390/toxins13110786 (PMC8619289; doi:10.3390/toxins13110786)
Supplement: Supplementary file 1 [file toxins-13-00786-s001.zip › FigureS3_Table_S3_Latin_America.pdf]

Supplementary Materials: Cyanotoxins and Food Contamination in Developing Countries: Review of Their Types, Toxicity, Analysis, Occurrence and Mitigation Strategies

Mohamed F. Abdallah, Wannes Van Hassel, Mirjana Andjelkovic, Annick Wilmotte and Andreja Rajkovic

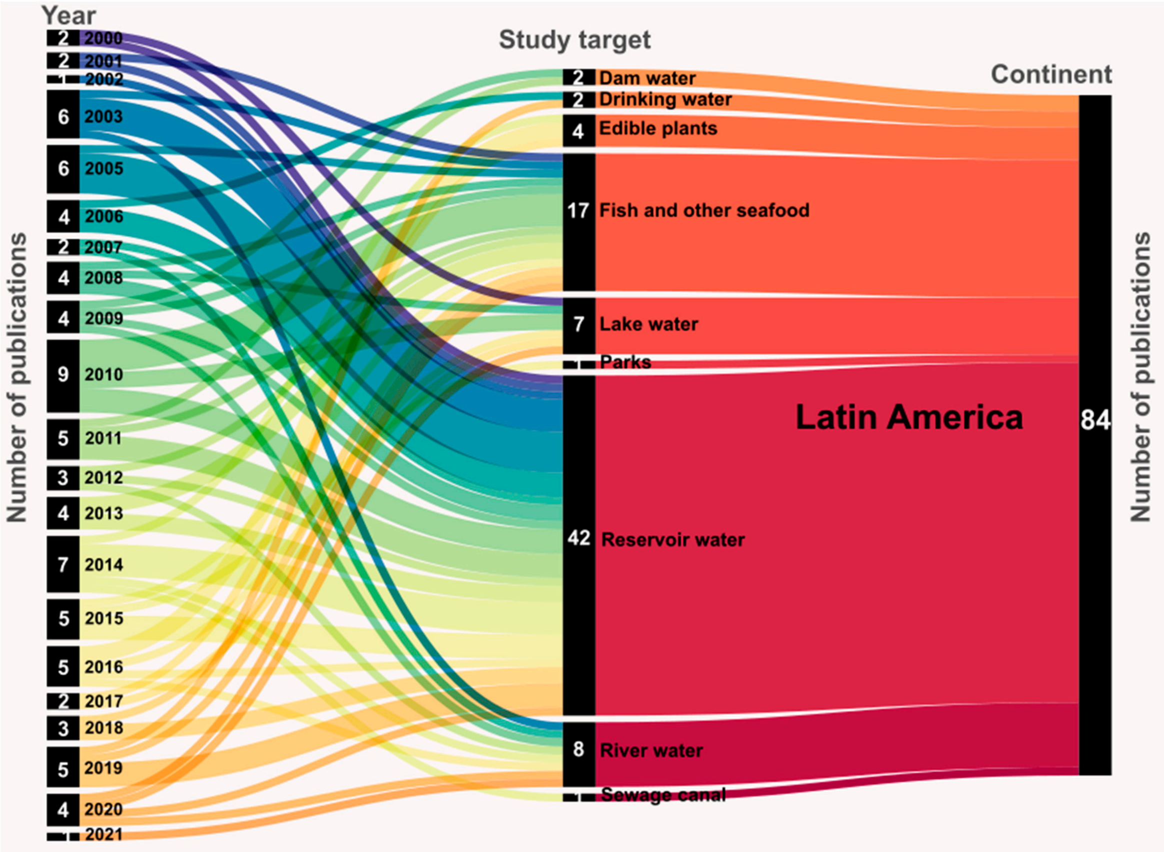

Figure S3. Number of articles published in each year between 2000 and October 2021 on the occurrence of cyanotoxin in different sources from the developing countries in Latin America.

Table S3. Number of publications focused on the natural occurrence of cyanotoxins in seafood as well as different environmental and water samples from Latin American developing countries between 2000 and 2021.

Country	Sample	Year	References
Argentina	Reservoir water	2003	[1]
Argentina	Reservoir water	2005	[2]
Argentina	Fish and other seafood	2010	[3]
Argentina	Fish and other seafood	2005	[4]
Argentina	Reservoir water	2005	[5]
Argentina	River water	2007	[6]
Argentina	River water	2016	[7]
Argentina	Fish and other seafood	2013	[8]
Argentina	Dam water	2011	[9]
Argentina	River water	2012	[10]
Argentina	Reservoir water	2010	[11]
Argentina	River water	2009	[12]
Argentina	Drinking water	2006	[13]
Argentina	Sewage canal	2014	[14]
Argentina	Reservoir water	2013	[15]
Argentina	River water	2014	[16]
Brazil	Reservoir water	2019	[17]
Brazil	Reservoir water	2010	[18]
Brazil	Reservoir water	2003	[19]
Brazil	Reservoir water	2005	[20]
Brazil	Reservoir water	2010	[21]
Brazil	Reservoir water	2011	[22]
Brazil	Reservoir water	2011	[21]
Brazil	Reservoir water	2014	[23]
Brazil	Edible plants	2016	[24]
Brazil	Reservoir water	2015	[25]
Brazil	Reservoir water	2007	[26]
Brazil	Lake water	2008	[27]
Brazil	Reservoir water	2003	[28]
Brazil	Reservoir water	2008	[29]
Brazil	Fish and other seafood	2010	[30]
Brazil	Edible plants	2016	[31]
Brazil	Edible plants	2017	[32]
Brazil	Reservoir water	2006	[33]
Brazil	Lake water	2010	[34]
Brazil	Fish and other seafood	2020	[35]
Brazil	Fish and other seafood	2008	[36]
Brazil	Parks	2015	[37]
Brazil	Dam water	2009	[38]
Brazil	Reservoir water	2015	[39]
Brazil	Fish and other seafood	2001	[40]
Brazil	Fish and other seafood	2009	[41]
Brazil	Reservoir water	2016	[42]
Brazil	Fish and other seafood	2015	[43]
Brazil	Fish and other seafood	2010	[44]
Brazil	Fish and other seafood	2018	[45]
Brazil	Reservoir water	2015	[46]

Brazil	Reservoir water	2018	[47]
Brazil	Reservoir water	2019	[48]
Brazil	Fish and other seafood	2003	[49]
Brazil	Reservoir water	2006	[50]
Brazil	Reservoir water	2003	[51]
Brazil	Reservoir water	2005	[52]
Brazil	Reservoir water	2000	[53]
Brazil	Reservoir water	2002	[54]
Brazil	Reservoir water	2005	[55]
Brazil	Reservoir water	2009	[56]
Brazil	Drinking water	2019	[57]
Brazil	Fish and other seafood	2013	[58]
Brazil	Reservoir water	2012	[59]
Brazil	Reservoir water	2013	[60]
Brazil	Reservoir water	2020	[61]
Brazil	Reservoir water	2011	[62]
Brazil	Reservoir water	2006	[63]
Brazil	Reservoir water	2008	[64]
Brazil	Reservoir water	2018	[65]
Brazil	River water	2003	[66]
Chile	Lake water	2016	[67]
Chile	Lake water	2000	[68]
Colombia	Reservoir water	2014	[69]
Guatemala	Edible plants	2014	[70]
Mexico	Reservoir water	2014	[71]
Mexico	Fish and other seafood	2010	[72]
Mexico	Fish and other seafood	2011	[73]
Mexico	Fish and other seafood	2012	[74]
Mexico	Lake water	2020	[75]
Mexico	Reservoir water	2014	[76]
Mexico	Reservoir water	2019	[77]
Mexico	Lake water	2010	[78]
Mexico	Lake water	2017	[79]
Mexico	Fish and other seafood	2019	[80]
Uruguay	River water	2020	[81]
Uruguay	Reservoir water	2001	[82]
Uruguay	River water	2021	[83]

References

1. Amé, M.V.; del Pilar Díaz, M.; Wunderlin, D.A. Occurrence of toxic cyanobacterial blooms in San Roque Reservoir (Córdoba, Argentina): A field and chemometric study. Environmental Toxicology 2003, 18, 192–201, doi:10.1002/tox.10114.
2. Amé, M.V.; Wunderlin, D.A. Effects of iron, ammonium and temperature on microcystin content by a natural concentrated Microcystis aeruginosa population. Water, Air, and Soil Pollution 2005, 168, 235–248, doi:10.1007/s11270-005-1774-8.
3. Amé, M.V.; Galanti, L.N.; Menone, M.L.; Gerpe, M.S.; Moreno, V.J.; Wunderlin, D.A. Microcystin-LR, -RR, -YR and -LA in water samples and fishes from a shallow lake in Argentina. Harmful Algae 2010, 9, 66–73, doi:10.1016/j.hal.2009.08.001.
4. Cazenave, J.; Wunderlin, D.A.; Bistoni, M.D.L.Á.; Amé, M.V.; Krause, E.; Pflugmacher, S.; Wiegand, C. Uptake, tissue distribution and accumulation of microcystin-RR in Corydoras paleatus, Jenynsia multidentata and Odontesthes bonariensis: A field and laboratory study. Aquatic Toxicology 2005, 75, 178–190, doi:10.1016/j.aquatox.2005.08.002.

5. Ruibal Conti, A.L.; Guerrero, J.M.; Regueira, J.M. Levels of microcystins in two Argentinean reservoirs used for water supply and recreation: Differences in the implementation of safe levels. Environmental Toxicology 2005, 20, 263–269, doi:10.1002/tox.20107.
6. Andrinolo, D.; Pereira, P.; Giannuzzi, L.; Aura, C.; Massera, S.; Caneo, M.; Caixach, J.; Barco, M.; Echenique, R. Occurrence of Microcystis aeruginosa and microcystins in Rio de la Plata river (Argentina). Acta Toxicológica Argentina 2007, 15, 8–14.
7. Forastier, M.E.; Zalocar, Y.; Andrinolo, D.; Domitrovic, H.A. Occurrence and toxicity of Microcystis aeruginosa (Cyanobacteria) in the Paraná River, downstream of the Yacyretá dam (Argentina). Revista de Biología Tropical 2016, 64, 219–227, doi:10.15517/rbt.v64i1.8993.
8. Galanti, L.N.; Amé, M.V.; Wunderlin, D.A. Accumulation and detoxification dynamic of cyanotoxins in the freshwater shrimp Palaemonetes argentinus. Harmful Algae 2013, 27, 88–97, doi:10.1016/j.hal.2013.05.007.
9. Giannuzzi, L.; Sedan, D.; Echenique, R.; Andrinolo, D. An acute case of intoxication with cyanobacteria and cyanotoxins in recreational water in Salto Grande Dam, Argentina. Marine Drugs 2011, 9, 2164–2175, doi:10.3390/md9112164.
10. Giannuzzi, L.; Carvajal, G.; Corradini, M.G.; Araujo Andrade, C.; Echenique, R.; Andrinolo, D. Occurrence of toxic cyanobacterial blooms in Rio de la Plata Estuary, Argentina: Field study and data analysis. Journal of Toxicology 2012, 2012, doi:10.1155/2012/373618.
11. Mancini, M.; Rodriguez, C.; Bagnis, G.; Liendo, A.; Prosperi, C.; Bonansea, M.; Tundisi, J.G. Cianobacterial bloom and animal mass mortality in a reservoir from Central Argentina. Brazilian Journal of Biology 2010, 70, 841–845, doi:10.1590/s1519-69842010000400015.
12. Otaño, S.H. First report of the cyanobacterium Aphanizomenon schindleri (Nostocales, Cyanophyceae) in River Uruguay, Argentina. Algological Studies 2009, 131, 35–42, doi:10.1127/1864-1318/2009/0131-0035.
13. Echenique, R.; Giannuzzi, L.; Ferrari, L. Drinking water: problems related to water supply in Bahía Blanca, Argentina. Acta toxicológica argentina 2006, 14, 23–30.
14. Rosso, L.; Sedan, D.; Kolman, M.; Caixach, J.; Flores, C.; Oteiza, J.M.; Salerno, G.; Echenique, R.; Giannuzzi, L.; Andrinolo, D. Microcystis aeruginos strain [D-Leu1] Mcyst-LR producer, from Buenos Aires province, Argentina. Journal of Coastal Life Medicine 2014, 2, 287–296, doi:10.12980/jclm.2.2014jclm-2014-0002.
15. Ruiz, M.; Galanti, L.; Ruibal, A.L.; Rodriguez, M.I.; Wunderlin, D.A.; Amé, M.V. First report of microcystins and anatoxin-a co-occurrence in San Roque reservoir (Córdoba, Argentina). Water, Air, and Soil Pollution 2013, 224, 1593, doi:10.1007/s11270-013-1593-2.
16. Sathicq, M.B.; Gómez, N.; Andrinolo, D.; Sedán, D.; Donadelli, J.L. Temporal distribution of cyanobacteria in the coast of a shallow temperate estuary (Río de la Plata): some implications for its monitoring. Environmental Monitoring and Assessment 2014, 186, 7115–7125, doi:10.1007/s10661-014-3914-3.
17. Barros, M.U.G.; Wilson, A.E.; Leitão, J.I.R.; Pereira, S.P.; Buley, R.P.; Fernandez-Figueroa, E.G.; Capelo-Neto, J. Environmental factors associated with toxic cyanobacterial blooms across 20 drinking water reservoirs in a semi-arid region of Brazil. Harmful Algae 2019, 86, 128–137, doi:10.1016/j.hal.2019.05.006.
18. Becker, V.; Ihara, P.; Yunes, J.S.; Huszar, V.L.M. Occurrence of anatoxin-a(s) during a bloom of Anabaena crassa in a water-supply reservoir in southern Brazil. Journal of Applied Phycology 2010, 22, 235–241, doi:10.1007/s10811-009-9451-8.
19. Bittencourt-Oliveira, M.D.C. Detection of potential microcystin-producing cyanobacteria in Brazilian reservoirs with a mcyB molecular marker. Harmful Algae 2003, 2, 51–60, doi:10.1016/S1568-9883(03)00004-0.
20. Bittencourt-Oliveira, M.D.C.; Kujbida, P.; Cardozo, K.H.M.; Carvalho, V.M.; Moura, A.D.N.; Colepicolo, P.; Pinto, E. A novel rhythm of microcystin biosynthesis is described in the cyanobacterium Microcystis panniformis Komárek et al. Biochemical and Biophysical Research Communications 2005, 326, 687–694, doi:10.1016/j.bbrc.2004.11.091.
21. Bittencourt-Oliveira, M. do C.; Piccin-Santos, V.; Gouvêa-Barros, S. Microcystin-producing genotypes from cyanobacteria in Brazilian reservoirs. Environmental Toxicology 2011, 27, 461–471, doi:10.1002/tox.20659.
22. Bittencourt-Oliveira, M. do C.; Piccin-Santos, V.; Kujbida, P.; Moura, A. do N. Cylindrospermopsin in Water Supply Reservoirs in Brazil Determined by Immunochemical and Molecular Methods. Journal of Water Resource and Protection 2011, 03, 349–355, doi:10.4236/jwarp.2011.36044.
23. Bittencourt-Oliveira, M.D.C.; Piccin-Santos, V.; Moura, A.N.; Aragão-Tavares, N.K.C.; Cordeiro-Araújo, M.K. Cyanobacteria, microcystins and cylindrospermopsin in public drinking supply reservoirs of Brazil. Anais da Academia Brasileira de Ciencias 2014, 86, 297–309, doi:10.1590/0001-3765201302512.
24. Bittencourt-Oliveira, M. do C.; Cordeiro-Araújo, M.K.; Chia, M.A.; Arruda-Neto, J.D. de T.; Oliveira, Ê.T. de; Santos, F. dos Lettuce irrigated with contaminated water: Photosynthetic effects, antioxidative response and bioaccumulation of microcystin congeners. Ecotoxicology and Environmental Safety 2016, 128, 83–90,

doi:10.1016/j.ecoenv.2016.02.014.
25. Borges, H.L.F.; Branco, L.H.Z.; Martins, M.D.; Lima, C.S.; Barbosa, P.T.; Lira, G.A.S.T.; Bittencourt-Oliveira, M.C.; Molica, R.J.R. Cyanotoxin production and phylogeny of benthic cyanobacterial strains isolated from the northeast of Brazil. Harmful Algae 2015, 43, 46–57, doi:10.1016/j.hal.2015.01.003.
26. De Carvalho, L.R.; Sant'Anna, C.L.; Gemelgo, M.C.P.; Azevedo, M.T.D.P. Cyanobacterial occurrence and detection of microcystin by planar chromatography in surface water of Billings and Guarapiranga Reservoirs, SP, Brazil. Revista Brasileira de Botanica 2007, 30, 141–148, doi:10.1590/S0100-84042007000100014.
27. De Carvalho, L.R.; Pipole, F.; Werner, V.R.; Laughinghouse IV, H.D.; De Camargo, A.C.M.; Rangel, M.; Konno, K.; Sant Anna, C.L. A toxic cyanobacterial bloom in an urban coastal lake, Rio Grande do Sul State, southern Brazil. Brazilian Journal of Microbiology 2008, 39, 761–769, doi:10.1590/S1517-83822008000400031.
28. Chellappa, N.T.; Mederios Costa, M.A. Dominant and co-existing species of Cyanobacteria from a Eutrophicated reservoir of Rio Grande do Norte State, Brazil. In Proceedings of the Acta Oecologica; Elsevier, 2003; Vol. 24, pp. S3–S10.
29. Chellappa, N.T.; Chellappa, S.L.; Chellappa, S. Harmful Phytoplankton Blooms and Fish Mortality in a eutrophicated reservoir of Northeast Brazil. 2008, 51, 833–841.
30. Clemente, Z.; Busato, R.H.; Oliveira Ribeiro, C.A.; Cestari, M.M.; Ramsdorf, W.A.; Magalhães, V.F.; Wosiack, A.C.; Silva de Assis, H.C. Analyses of paralytic shellfish toxins and biomarkers in a southern Brazilian reservoir. Toxicon 2010, 55, 396–406, doi:10.1016/j.toxicon.2009.09.003.
31. Cordeiro-Araújo, M.K.; Chia, M.A.; Arruda-Neto, J.D. de T.; Tornisielo, V.L.; Vilca, F.Z.; Bittencourt-Oliveira, M. do C. Microcystin-LR bioaccumulation and depuration kinetics in lettuce and arugula: Human health risk assessment. Science of the Total Environment 2016, 566–567, 1379–1386, doi:10.1016/j.scitotenv.2016.05.204.
32. Cordeiro-Araújo, M.K.; Chia, M.A.; Bittencourt-Oliveira, M. do C. Potential human health risk assessment of cylindrospermopsin accumulation and depuration in lettuce and arugula. Harmful Algae 2017, 68, 217–223, doi:10.1016/j.hal.2017.08.010.
33. Costa, I.A.S.; Azevedo, S.M.F.O.; Senna, P.A.C.; Bernardo, R.R.; Costa, S.M.; Chellappa, N.T. Occurrence of toxin-producing cyanobacteria blooms in a Brazilian semiarid reservoir. Brazilian Journal of Biology 2006, 66, 211–219, doi:10.1590/S1519-69842006000200005.
34. da Silva, R.R.P.; Pires, O.R.; Grisolia, C.K. Toxicity and genotoxicity in Astyanax bimaculatus (Characidae) induced by microcystins from a bloom of Microcystis spp. Genetics and Molecular Biology 2010, 33, 750–755, doi:10.1590/S1415-47572010000400023.
35. De Bock, M.F.S.; Moraes, G.S. de O.; Almeida, R.G. dos S.; Vieira, K.D. da S.; Santoro, K.R.; Bicudo, Á.J. de A.; Molica, R.J.R. Exposure of Nile Tilapia (Oreochromis niloticus) Fingerlings to a Saxitoxin-Producing Strain of Raphidiopsis (Cylindrospermopsis) raciborskii (Cyanobacterium) Reduces Growth Performance and Increases Mortality Rate. Environmental Toxicology and Chemistry 2020, 39, 1409–1420, doi:10.1002/etc.4728.
36. Deblois, C.P.; Aranda-Rodriguez, R.; Giani, A.; Bird, D.F. Microcystin accumulation in liver and muscle of tilapia in two large Brazilian hydroelectric reservoirs. Toxicon 2008, 51, 435–448, doi:10.1016/j.toxicon.2007.10.017.
37. Elias, L.M.; Silva-Stenico, M.E.; Alvarenga, D.O.; Rigonato, J.; Fiore, M.F.; Lira, S.P. de Molecular and Chemical Analyses of Cyanobacterial Blooms in Tropical Lagoons from Southeast Brazil. Journal of Water Resource and Protection 2015, 07, 50–71, doi:10.4236/jwarp.2015.71004.
38. Fiore, M.F.; Genuário, D.B.; da Silva, C.S.P.; Shishido, T.K.; Moraes, L.A.B.; Neto, R.C.; Silva-Stenico, M.E. Microcystin production by a freshwater spring cyanobacterium of the genus Fischerella. Toxicon 2009, 53, 754–761, doi:10.1016/j.toxicon.2009.02.010.
39. Fonseca, J.R.; Vieira, P.C.S.; Kujbida, P.; Soares Da Costa, I.A. Cyanobacterial occurrence and detection of microcystins and saxitoxins in reservoirs of the Brazilian semi-arid. Acta Limnologica Brasiliensia 2015, 27, 78–92, doi:10.1590/S2179-975X2814.
40. Freitas de Magalhães, V.; Moraes Soares, R.; Azevedo, S.M.F.O. Microcystin contamination in fish from the Jacarepaguá Lagoon (Rio de Janeiro, Brazil): Ecological implication and human health risk. Toxicon 2001, 39, 1077–1085, doi:10.1016/S0041-0101(00)00251-8.
41. Galvão, J.A.; Oetterer, M.; Bittencourt-Oliveira, M. do C.; Gouvêa-Barros, S.; Hiller, S.; Erler, K.; Luckas, B.; Pinto, E.; Kujbida, P. Saxitoxins accumulation by freshwater tilapia (Oreochromis niloticus) for human consumption. Toxicon 2009, 54, 891–894, doi:10.1016/j.toxicon.2009.06.021.
42. Genuário, D.B.; Lorenzi, A.S.; Agujaro, L.F.; Isaac, R. de L.; Azevedo, M.T. de P.; Cantúsio Neto, R.; Fiore,

M.F. Cyanobacterial community and microcystin production in a recreational reservoir with constant Microcystis blooms. Hydrobiologia 2016, 779, 105–125, doi:10.1007/s10750-016-2802-y.

43. Hauser-Davis, R.A.; Lavradas, R.T.; Lavandier, R.C.; Rojas, E.G.A.; Guarino, A.W.S.; Ziolli, R.L. Accumulation and toxic effects of microcystin in tilapia (Oreochromis niloticus) from an eutrophic Brazilian lagoon. Ecotoxicology and Environmental Safety 2015, 112, 132–136, doi:10.1016/j.ecoenv.2014.10.036.
44. Leão, J. de C.; Giordano, S.B.; Yunes, J.S. Microcystins uptake by the yellow clam Mesodesma mactroides (BIVALVIA, MACTROIDEA). Atlântica 2010, 32, 79–85, doi:10.5088/atl.2010.32.1.79.
45. Calado, S.L. de M.; Santos, G.S.; Leite, T.P.B.; Wojciechowski, J.; Nadaline, M.; Bozza, D.C.; Magalhães, V.F. de; Cestari, M.M.; Prodocimo, V.; Silva de Assis, H.C. Depuration time and sublethal effects of microcystins in a freshwater fish from water supply reservoir. Chemosphere 2018, 210, 805–815, doi:10.1016/j.chemosphere.2018.07.075.
46. Lorenzi, A.S.; Chia, M.A.; Piccin-Santos, V.; Bittencourt-Oliveira, M.D.C. Microcystins and cylindrospermopsins molecular markers for the detection of toxic cyanobacteria: A case study of northeastern Brazilian reservoirs. Limnetica 2015, 34, 269–282.
47. Lorenzi, A.S.; Cordeiro-Araújo, M.K.; Chia, M.A.; Bittencourt-Oliveira, M. do C. Cyanotoxin contamination of semiarid drinking water supply reservoirs. Environmental Earth Sciences 2018, 77, 1–8, doi:10.1007/s12665-018-7774-y.
48. Lorenzi, A.S.; Chia, M.A.; Lopes, F.A.C.; Silva, G.G.Z.; Edwards, R.A.; Bittencourt-Oliveira, M. do C. Cyanobacterial biodiversity of semiarid public drinking water supply reservoirs assessed via next-generation DNA sequencing technology. Journal of Microbiology 2019, 57, 450–460, doi:10.1007/s12275-019-8349-7.
49. Magalhães, V.F.; Marinho, M.M.; Domingos, P.; Oliveira, A.C.; Costa, S.M.; Azevedo, L.O.; Azevedo, S.M.F.O. Microcystins (cyanobacteria hepatotoxins) bioaccumulation in fish and crustaceans from Sepetiba Bay (Brasil, RJ). Toxicon 2003, 42, 289–295, doi:10.1016/S0041-0101(03)00144-2.
50. Anjos, F.M. dos; Bittencourt-Oliveira, M. do C.; Zajac, M.P.; Hiller, S.; Christian, B.; Erler, K.; Luckas, B.; Pinto, E. Detection of harmful cyanobacteria and their toxins by both PCR amplification and LC-MS during a bloom event. Toxicon 2006, 48, 239–245, doi:10.1016/j.toxicon.2006.05.006.
51. Vieira, J.M.D.S.; Azevedo, M.T.D.P.; De Oliveira Azevedo, S.M.F.; Honda, R.Y.; Corrêa, B. Microcystin production by Radiocystis fernandoi (Chroococcales, Cyanobacteria) isolated from a drinking water reservoir in the city of Belém, PA, Brazilian Amazonia region. Toxicon 2003, 42, 709–713, doi:10.1016/j.toxicon.2003.08.004.
52. Vieira, J.M.D.S.; Azevedo, M.T.D.P.; De Oliveira Azevedo, S.M.F.; Honda, R.Y.; Corrêa, B. Toxic cyanobacteria and microcystin concentrations in a public water supply reservoir in the Brazilian Amazonia region. Toxicon 2005, 45, 901–909, doi:10.1016/j.toxicon.2005.02.008.
53. Matthiensen, A.; Beattie, K.A.; Yunes, J.S.; Kaya, K.; Codd, G.A. [D-Leu1]Microcystin-LR, from the cyanobacterium Microcystis RST 9501 and from a Microcystis bloom in the Patos Lagoon estuary, Brazil. Phytochemistry 2000, 55, 383–387, doi:10.1016/S0031-9422(00)00335-6.
54. Mollcai, R.; Onodera, H.; Garcfa, C.; Rivas, M.; Andrinol, D.; Nascimentolt, S.; Megur, H.; Oshima, Y.; Azeved, S.; Lagos, N. Toxins in the freshwater cyanobacterium Cylindrospermopsis raciborskii (Cyanophyceae) isolated from Tabocas reservoir in Caruaru, Brazil, including demonstration of a new saxitoxin analogue. Phycologia 2002, 41, 606–611.
55. Molica, R.J.R.; Oliveira, E.J.A.; Carvalho, P.V.V.C.; Costa, A.N.S.F.; Cunha, M.C.C.; Melo, G.L.; Azevedo, S.M.F.O. Occurrence of saxitoxins and an anatoxin-a(s)-like anticholinesterase in a Brazilian drinking water supply. Harmful Algae 2005, 4, 743–753, doi:10.1016/j.hal.2004.11.001.
56. Moschini-Carlos, V.; Bortoli, S.; Pinto, E.; Nishimura, P.Y.; De Freitas, L.G.; Pompêo, M.L.M.; Dörr, F. Cyanobacteria and cyanotoxin in the billings reservoir (São Paulo, SP, Brazil). Limnetica 2009, 28, 273–282.
57. Oliveira, E.D.C.; Castelo-Branco, R.; Silva, L.; Silva, N.; Azevedo, J.; Vasconcelos, V.; Faustino, S.; Cunha, A. First detection of microcystin-lr in the amazon river at the drinking water treatment plant of the municipality of Macapá, Brazil. Toxins 2019, 11, 1–21, doi:10.3390/toxins11110669.
58. Oliveira, N.B.; Schwartz, C.A.; Bloch, C.; Paulino, L.; Pires, O.R. Bioacumulation of cyanotoxins in hypophthalmichthys molitrix (silver carp) in paranoá lake, Brasilia-DF, Brazil. Bulletin of Environmental Contamination and Toxicology 2013, 90, 308–313, doi:10.1007/s00128-012-0873-7.
59. Piccin-Santos, V.; Bittencourt-Oliveira, M. do C. Toxic Cyanobacteria in Four Brazilian Water Supply Reservoirs. Journal of Environmental Protection 2012, 03, 68–73, doi:10.4236/jep.2012.31009.
60. Pimentel, J.S.M.; Giani, A. Estimating toxic cyanobacteria in a Brazilian reservoir by quantitative real-time PCR, based on the microcystin synthetase D gene. Journal of Applied Phycology 2013, 25, 1545–1554, doi:10.1007/s10811-013-9996-4.

61. Rego, A.H.G.; Rangel-Junior, A.; Costa, I.A.S. Phytoplankton scenario and microcystin in water during extreme drought in semiarid tropical water supplies, Northeastern Brazil. Brazilian Journal of Biology 2020, 80, 1–11, doi:10.1590/1519-6984.182599.
62. Sant'Anna, C.L.; De Carvalho, L.R.; Fiore, M.F.; Silva-Stenico, M.E.; Lorenzi, A.S.; Rios, F.R.; Konno, K.; Garcia, C.; Lagos, N. Highly toxic microcystis aeruginosa strain, isolated from São Paulo-Brazil, produce hepatotoxins and paralytic shellfish poison neurotoxins. Neurotoxicity Research 2011, 19, 389–402, doi:10.1007/s12640-010-9177-z.
63. Sotero-Santos, R.B.; Silva, C.R.D.S.E.; Verani, N.F.; Nonaka, K.O.; Rocha, O. Toxicity of a cyanobacteria bloom in Barra Bonita Reservoir (Middle Tietê River, São Paulo, Brazil). Ecotoxicology and Environmental Safety 2006, 64, 163–170, doi:10.1016/j.ecoenv.2005.03.011.
64. Sotero-Santos, R.B.; Carvalho, E.G.; Dellamano-Oliveira, M.J.; Rocha, O. Occurrence and toxicity of an Anabaena bloom in a tropical reservoir (Southeast Brazil). Harmful Algae 2008, 7, 590–598, doi:10.1016/j.hal.2007.12.017.
65. Walter, J.M.; Lopes, F.A.C.; Lopes-Ferreira, M.; Vidal, L.M.; Leomil, L.; Melo, F.; de Azevedo, G.S.; Oliveira, R.M.S.; Medeiros, A.J.; Melo, A.S.O.; et al. Occurrence of harmful cyanobacteria in drinking water from a severely drought-impacted semi-arid region. Frontiers in Microbiology 2018, 9, doi:10.3389/fmicb.2018.00176.
66. Yunes, J.S.; Cunha, N.T.; Barros, L.P.; Proença, L.A.O.; Monserrat, J.M. Cyanobacterial Neurotoxins from Southern Brazilian Freshwaters. Comments on Toxicology 2003, 9, 103–115, doi:10.1080/08865140302426.
67. Almanza, V.; Parra, O.; De Carlos Bicudo, C.E.; Baeza, C.; Beltran, J.; Figueroa, R.; Urrutia, R. Occurrence of toxic blooms of Microcystis aeruginosa in a central Chilean (36° Lat. S) urban lake. Revista Chilena de Historia Natural 2016, 89, 8, doi:10.1186/s40693-016-0057-7.
68. Neumann, U.; Campos, V.; Cantarero, S.; Urrutia, H.; Heinze, R.; Weckesser, J.; Erhard, M. Co-occurrence of non-toxic (Cyanopeptolin) and toxic (Microcystin) peptides in a bloom of Microcystis sp. fuom a Chilean lake. Systematic and Applied Microbiology 2000, 23, 191–197, doi:10.1016/S0723-2020(00)80004-1.
69. Ferrão-Filho, A.S.; Herrera, N.A.; Echeverri, L.F. Microcystin accumulation in cladocerans: First evidence of MC uptake from aqueous extracts of a natural bloom sample. Toxicon 2014, 87, 26–31, doi:10.1016/j.toxicon.2014.05.015.
70. Romero-Oliva, C.S.; Contardo-Jara, V.; Block, T.; Pflugmacher, S. Accumulation of microcystin congeners in different aquatic plants and crops - A case study from lake Amatitlán, Guatemala. Ecotoxicology and Environmental Safety 2014, 102, 121–128, doi:10.1016/j.ecoenv.2014.01.031.
71. Alillo-Sánchez, J.L.; Gaytán-Herrera, M.L.; Martínez-Almeida, V.M.; Ramírez-García, P. Microcystin-LR equivalents and their correlation with Anabaena spp. in the main reservoir of a hydraulic system of Central Mexico. Inland Waters 2014, 4, 327–336, doi:10.5268/IW-4.3.573.
72. Berry, J.P.; Lind, O. First evidence of "paralytic shellfish toxins" and cylindrospermopsin in a Mexican freshwater system, Lago Catemaco, and apparent bioaccumulation of the toxins in "tegogolo" snails (Pomacea patula catemacensis). Toxicon 2010, 55, 930–938, doi:10.1016/j.toxicon.2009.07.035.
73. Berry, J.P.; Lee, E.; Walton, K.; Wilson, A.E.; Bernal-Brooks, F. Bioaccumulation of microcystins by fish associated with a persistent cyanobacterial bloom in Lago de Patzcuaro (Michoacan, Mexico). Environmental Toxicology and Chemistry 2011, 30, 1621–1628, doi:10.1002/etc.548.
74. Berry, J.P.; Jaja-Chimedza, A.; Dávalos-Lind, L.; Lind, O. Apparent bioaccumulation of cylindrospermopsin and paralytic shellfish toxins by finfish in lake catemaco (Veracruz, Mexico). Food Additives and Contaminants - Part A Chemistry, Analysis, Control, Exposure and Risk Assessment 2012, 29, 314–321, doi:10.1080/19440049.2011.597785.
75. Bustillos-Guzmán, J.J.; Turner, A.; Hernández-Almeida, O.U.; Band-Schmidt, C.J.; Romero-Bañuelos, C.A.; Hernández-Sandoval, F.E.; Núñez-Vázquez, E.J.; Palomino-Hermosillo, Y.A. Presence of cyanotoxins in a mexican subtropical monomictic crater lake. Applied Sciences (Switzerland) 2020, 10, 1–15, doi:10.3390/app10196719.
76. Figueroa-Sanchez, M.A.; Nandini, S.; Sarma, S.S.S. Zooplankton community structure in the presence of low levels of cyanotoxins: A case study in a high altitude tropical reservoir (Valle de Bravo, Mexico). Journal of Limnology 2014, 73, 157–166, doi:10.4081/jlimnol.2014.784.
77. Nandini, S.; Sánchez-Zamora, C.; Sarma, S.S.S. Toxicity of cyanobacterial blooms from the reservoir Valle de Bravo (Mexico): A case study on the rotifer Brachionus calyciflorus. Science of the Total Environment 2019, 688, 1348–1358, doi:10.1016/j.scitotenv.2019.06.297.
78. Vasconcelos, V.; Martins, A.; Vale, M.; Antunes, A.; Azevedo, J.; Welker, M.; Lopez, O.; Montejano, G. First report on the occurrence of microcystins in planktonic cyanobacteria from Central Mexico. Toxicon 2010,

56, 425–431, doi:10.1016/j.toxicon.2010.04.011.
79. Barrios, C.A.Z.; Nandini, S.; Sarma, S.S.S. Effect of crude extracts from cyanobacterial blooms in Lake Texcoco (Mexico) on the population growth of Brachionus calyciflorus (Rotifera). Toxicon 2017, 139, 45–53, doi:10.1016/j.toxicon.2017.09.013.
80. Zamora-Barrios, C.A.; Nandini, S.; Sarma, S.S.S. Bioaccumulation of microcystins in seston, zooplankton and fish: A case study in Lake Zumpango, Mexico. Environmental Pollution 2019, 249, 267–276, doi:10.1016/j.envpol.2019.03.029.
81. Aubriot, L.; Zabaleta, B.; Bordet, F.; Sienra, D.; Risso, J.; Achkar, M.; Somma, A. Assessing the origin of a massive cyanobacterial bloom in the Río de la Plata (2019): Towards an early warning system. Water Research 2020, 181, 115944, doi:10.1016/j.watres.2020.115944.
82. De Leon, L.; Yunes, J.S. First report of a Microcystin-containing bloom of the cyanobacterium Microcystis aeruginosa in the La Plata River, South America. Environmental Toxicology 2001, 16, 110–112, doi:10.1002/1522-7278(2001)16:1<110::AID-TOX1012>3.0.CO;2-Z.
83. Kruk, C.; Martínez, A.; Martínez de la Escalera, G.; Trinchin, R.; Manta, G.; Segura, A.M.; Piccini, C.; Brena, B.; Yannicelli, B.; Fabiano, G.; et al. Rapid freshwater discharge on the coastal ocean as a mean of long distance spreading of an unprecedented toxic cyanobacteria bloom. Science of the Total Environment 2021, 754, 142362, doi:10.1016/j.scitotenv.2020.142362.
